# Myeloid ZNRF1 suppresses autoimmune demyelination and neuroinflammation by regulating MHC-II-mediated T cell activation

**DOI:** 10.1186/s12974-025-03550-z

**Published:** 2025-10-22

**Authors:** Yung-Chi Chang, Ching-Chih Wu, Fu-Ting Hsiao, You-Sheng Lin, Ting-Yu Lai, Kang-Hsuan Huang, Shiue-Cheng Tang, Chih-Yuan Lee, Shen-Ju Chou, Li-Chung Hsu

**Affiliations:** 1 Institute of Molecular Medicine, College of Medicine, National Taiwan University, Taipei, Taiwan; 2 Institute of Biotechnology, National Tsing Hua University, Hsinchu, Taiwan; 3 Department of Surgery, National Taiwan University Hospital, Taipei, Taiwan; 4 Institute of Cellular and Organismic Biology, Academia Sinica, Taipei, Taiwan; 5 Graduate Institute of Immunology, College of Medicine, National Taiwan University, Taipei, Taiwan

**Keywords:** Multiple Sclerosis, Neuroinflammation, Experimental autoimmune encephalomyelitis, Macrophage, ZNRF1

## Abstract

Multiple sclerosis (MS) is an autoimmune and neuroinflammation disease characterized by axonal damage, inflammatory demyelination, and neurodegeneration. However, the precise mechanisms underlying MS pathogenesis remain largely unclear. Here, we identify ZNRF1, an E3 ubiquitin ligase, as a critical regulator of experimental autoimmune encephalomyelitis (EAE), a murine model that recapitulates the autoimmune demyelination features of MS. Mice lacking ZNRF1 exhibit increased susceptibility to EAE progression. Notably, ZNRF1 depletion in peripheral myeloid cells, but not in microglia, leads to enhanced immune cell infiltration into the central nervous system, resulting in demyelination and exacerbated disease severity. The heightened EAE severity in *Znrf1*-deficient mice is associated with increased polarization of Th1 and Th17 cells, elevated antigen-specific T helper cell proliferation, and amplified immune responses. Furthermore, following EAE induction, macrophages from *Znrf1*-deficient mice display elevated surface expression of MHC class II (MHC-II) molecules. Collectively, our findings suggest that ZNRF1 in peripheral myeloid cells plays a suppressive role in neuroinflammation by regulating MHC-II surface expression, thereby controlling antigen-specific T-cell proliferation and activation.

**Supplementary Information:**

The online version contains supplementary material available at 10.1186/s12974-025-03550-z.

## Introduction

Multiple sclerosis (MS) is a chronic autoimmune disorder of the central nervous system (CNS) that primarily targets myelinated axons [12, 13, 23, 57]. The immune system erroneously attacks the myelin sheath surrounding nerve fibers, leading to inflammation, demyelination, and the formation of lesions (plaques), the pathological hallmark of MS, with in gray and white matter of the brain, spinal cord, and optic nerve [26, 66]. Although the etiology of MS remains poorly understood, both genetic and environmental factors are believed to contribute to its development [2, 23, 35, 63]. The pathophysiology of MS involves intricate interactions between the innate and adaptive immune systems, with peripheral immune cells such as B cells, T cells, and myeloid cells (e.g., dendritic cells (DCs) and macrophages), as well as CNS-resident cells including microglia and astrocytes, playing pivotal roles [1, 5, 7, 19, 23, 33, 38, 41, 55, 62, 73, 75]. However, despite extensive research, the precise mechanisms underlying MS pathogenesis remain elusive.

CD4^+^ T cells are central to the pathogenesis of MS [5, 19, 30, 45, 53]. Autoreactive CD4^+^ T cells activate B cells and differentiate into various subsets of T helper (Th) cells, which orchestrate immune responses. Naive T cell activation requires antigen presentation by antigen-presenting cells (APCs), such as B cells, macrophages, and DCs [61]. In MS patients, elevated numbers of interferon (IFN)-γ-producing CD4^+^ T (Th1) cells, interleukin-17 (IL-17)-producing CD4^+^ T (Th17) cells, and cytotoxic CD8^+^ T cells are found in both peripheral blood and the CNS, contributing to oligodendrocyte and neuronal damage [52, 53]. Activated CD4^+^ T cells proliferate, differentiate into specific Th-cell subsets, and generate memory T cells [19, 69], which are categorized into central memory T (T_CM_) cells and effector memory T (T_EM_) cells [6, 69]. In MS patients, autoreactive CD4^+^ T_EM_ cells are enriched, whereas T_CM_ cells are reduced in peripheral blood compared to healthy individuals [44], highlighting the importance of T-cell subset balance in MS pathophysiology.

APCs critically shape T-cell responses in MS. Monocytes differentiate into macrophages that express major histocompatibility complex class II (MHC-II) and costimulatory molecules such as CD80 and CD86 and secrete proinflammatory cytokines that drive CNS demyelination [1, 29, 41, 87, 89]. Additionally, monocyte infiltration into lesions correlates with disease severity during early MS [1, 41, 80]. DCs, as professional APCs, initiate both innate and adaptive immune responses [25, 62, 75]. They are broadly categorized into conventional DCs (cDCs), which express high levels of MHC-II to present antigen, and plasmacytoid DCs (pDCs), which primarily produce robust type I interferons in response to viral infections [5, 25]. Notably, cDCs are elevated in the blood of patients with secondary progressive MS (SPMS) compared to relapsing–remitting MS (RRMS) and healthy controls [5, 38]. APCs fine-tune T-cell activation through stimulatory and inhibitory pathways: MHC-II promotes CD4^+^ T-cells activation, whereas inhibitory molecules such as programmed cell death protein-1 (PD-1) and its ligand programmed cell death ligand-1 (PD-L1), as well as Fas and its ligand FasL regulate T-cell apoptosis and tolerance. Dysregulation of these pathways are associated with MS pathogenesis [9, 20, 24, 28, 42, 43, 48, 64, 82, 84].

Microglia, the resident macrophages of the CNS, also play dual roles in MS pathology [87, 92]. During early disease stages, activated microglia contribute to the formation of demyelinating lesions by secreting proinflammatory cytokines and chemokines, which recruit T cells into the CNS, thereby accelerating MS pathophysiology [36, 59, 76, 92]. Conversely, at later stages, microglia mediate neuroprotection through clearance of myelin debris, secretion of anti-inflammatory cytokines and neuroprotective factors, and enhancement of antioxidant responses that promote remyelination [9, 46, 79]. These context-dependent roles highlight the functional plasticity of microglia in MS progression.

ZNRF1, an E3 ubiquitin ligases, contains zinc and ring finger domains essential for its E3 ligase activity [3, 4]. ZNRF1 also possesses an N-myristoylated domain and is localized to the endosome–lysosome compartments [4]. Under oxidative stress conditions, such as neuronal apoptosis and axonal degeneration, ZNRF1 activity is enhanced by epidermal growth factor receptor (EGFR)-mediated phosphorylation at tyrosine 103, leading to ubiquitination and degradation of protein kinase B (AKT) and promoting Wallerian degeneration [85, 86]. Beyond neuronal function, ZNRF1 plays significant roles in innate immune regulation. Our recent work shows that ZNRF1 ubiquitinates and degrades caveolin-1 through AKT-GSK3β signaling, thereby promoting Toll-like receptor 4 (TLR4)-mediated inflammatory responses essential for host defense against gram-negative bacteria [47]. Additionally, we have demonstrated that c-Src-activated ZNRF1 negatively regulates TLR3 signaling by facilitating lysosomal degradation of TLR3 [50]. ZNRF1 also targets EGFR for degradation, modulating its downstream signaling [74]. These findings highlight the multifaceted role of ZNRF1 in both neuronal and immune system regulation. Given this background, we hypothesize that ZNRF1 may influence MS pathogenesis through its effects on immune cell function and neuroinflammation. In the present study, we investigate the role of ZNRF1 in various immune cells involved in EAE. Our findings suggest that ZNRF1 in peripheral myeloid cells, but not microglia, controls the surface expression of MHC-II, thereby modulating antigen-specific T-cell proliferation and activation. These results underscore a previously unrecognized immunosuppressive role for ZNRF1 in myeloid cells during neuroinflammatory responses.

## Materials and methods

### Mice

*Znrf1*^F/F^ and *Znrf1*^−/−^ mice were previously described [47, 50]. To generate a myeloid cell lineage with *Znrf1* deletion (referred to as *Znrf1*^Δmye^), *Znrf1*^F/F^ mice were crossed with *LysM*-*Cre* mice (004781; The Jackson Laboratory). To create a CNS tissue-resident microglial lineage with *Znrf1* deletion (*Znrf1*^Δglia^), *Znrf1*^F/F^ mice were crossed with *Cx3cr1-CreERT2*-EYFP mice (021160; The Jackson Laboratory). Microglial *Znrf1* gene deletion was induced by intraperitoneally injecting the mice with tamoxifen (0.135 mg/g body weight) for 5 consecutive days. A subsequent waiting period of 4–6 weeks was allowed to ensure replenishment of peripheral wild-type macrophages. All animals were housed in a pathogen-free animal facility, and all animal experiments were performed in accordance with animal welfare guidelines, approved by the Institutional Animal Care and Use Committee of the College of Medicine, National Taiwan University (approval number: 20180450 and 20230070).

### Antibodies and reagents

CD4-BV711 (#563726; clone: RM4-5; dilution, 1:1000), IL-17A-BV421 (#563354; clone, TC11-18H10; dilution, 1:500), H-2 Kb/H-2Db-BV480 (#746258; clone, C3H.SW; dilution, 1:400), RORγt-PE (#562607; clone, Q31-378; dilution, 1:100) and Brilliant Stain Buffer Plus (#566385) were from BD Pharmingen (Basel, Switzerland). CD80-PE-Cy7 (#60–0801; clone:16-10A1; dilution, 1:400) and Ly-6G-violetFluor 450 (#75–1276; clone, 1A8; dilution, 1:200) were obtained from Cytek Biosciences (San Diego, CA). CD11b-PE-Cy7 (#101216; clone, M1/70; dilution, 1:100), CD11b-BV510 (#101263; clone, M1/70; dilution, 1:800), CD11c-APC (#117310; clone, N418; dilution, 1:1000), CD11c-PE (#117308; clone, N418; dilution, 1:1000), CD172a-PE-Dazzle 594 (#144016; clone, P84; dilution, 1:200), CD178 (FasL)-PE (#106605; clone, MFL3; dilution, 1:100), CD19-PE (#152407; clone, 1D3; dilution, 1:1000), CD25-BV421 (#102043; clone, PC61; dilution, 1:500), CD274 (B7-H1, PD-L1)-BV421 (#124315; clone, 10F.9G2; dilution, 1:200), CD3-APC (#100322; clone, 145-2C11; dilution, 1:200), CD3-PE (#100205; clone, 17A2; dilution, 1:1000), CD44-APC (#103012; clone, IM7; dilution, 1:1000), CD45-FITC (#103108; clone, 30-F11; dilution, 1:1000), CD40-APC/Cyanine 7 (#124638; clone, 3/23; dilution, 1:100), CD45-BV421 (#103147; clone, 30-F11; dilution, 1:1000), CD45-PerCP-Cy5.5 (#103132; clone, 30-F11; dilution, 1:800), CD45R/B220-FITC (#103206; clone, RA3-6B2; dilution, 1:500), CD45R/B220-Alexa Fluor 700 (#103232; clone, RA3-6B2; dilution, 1:100), CD62L-BV605 (#104438; clone, MEL-14; dilution, 1:1000), CD69-PE (#104507; clone, H1.2F3; dilution, 1:500), CD8a-PE (#100708; clone, 53–6.7; dilution, 1:1000), CD86-PE/Cy 5 (#105015; clone, GL-1; dilution, 1:400), Gr1 (Ly-6G/Ly-6C)-PE (#108407; clone, RB6-8C5; dilution, 1:1000), F4/80-BV711 (#123147; clone, BM8; dilution, 1:100), I-A/I-E (MHC-II)-BV605 (#107639; clone, M5/114.15.2; dilution, 1:400), IFN-γ-APC (#505810; clone, XMG1.2; dilution, 1:500), Ly-6C-FITC (#128006; clone, HK1.4; dilution, 1:400), T-bet-BV605 (#644817; clone, 4B10, dilution, 1:100) and XCR1-BV785 (#148225; clone, ZET; dilution, 1:100) were purchased from Biolegend (San Diego, CA).

### Establishment of the Experimental Autoimmune Encephalomyelitis (EAE) model

To establish a mouse model of EAE, mice aged 8–12 weeks were immunized with 200 µg of MOG_35–55_ (sequence: MEVGWYRSPFSRVVHLYRNGK; #SC1208; GeneScript, Piscataway, NJ) in 100 µL of 1X Dulbecco’s PBS (DPBS; Gibco, Waltham, MA), mixed with 100 µL of Freund’s complete adjuvant (CFA; #263810; BD/DIFICO, Franklin Lakes, NJ), containing 4 mg of *Mycobacterium tuberculosis* H37Ra (#231141, BD/DIFCO). Mice received two subcutaneous injections of 100 µL of MOG_35-55_/CFA emulsion on day 0. Additionally, they were intraperitoneally injected with 200 ng of pertussis toxin (#180, List Biological Laboratories, Campbell, CA) in 500 µL of 1X DPBS on days 0, 2, and 7. The mice were monitored for weight changes and clinical symptoms, which were scored using the following scale: grade 0, normal with no observable neurological defect; grade 0.5, partial paralysis of the tail; grade 1, complete paralysis of the tail; grade 2, impaired righting reflex; grade 2.5, impaired movement coordination; grade 3, abnormal gait; grade 3.5, partial paresis of the hindlimbs; grade 4, paresis affecting both hindlimbs; grade 4.5, able to paddle with both hindlimbs, but neither hindlimb able to move forward past the hip joint; grade 5, paralysis of one hindlimb; grade 5.5, complete paralysis of both hindlimbs; grade 6, complete paralysis of both hindlimbs with labored breathing; grade 6.5, paralysis of both hindlimbs with partial paralysis of forelimbs or moribund state; grade 7, death. Intermediate values (in increments of 0.25) were used to reflect gradual symptom progression between these defined points.

### Isolation of CNS-infiltrating cells

Mice were perfused with 40 mL of ice-cold 1X DPBS to remove blood from circulation, followed by the collection of brain and spinal cord tissues. The collected tissues were minced and digested enzymatically for 45 min at 37 °C with rotation at 250 RPM in a shaker using a solution of 0.1 mg/mL collagenase/dispase (#10269638001, Roche, Basel, Switzerland) and 0.75 mg/mL DNase I (#10104159001; Roche) in Roswell Park Memorial Institute (RPMI) medium (Gibco) containing 2% fetal bovine serum (FBS; Corning, Woodland, CA), 1% non-essential amino acids (NEAA; Gibco), 1% L-glutamine (Sartorius, Beit Haemek, Israel), 1% penicillin–streptomycin (Sartorius), and 1% sodium pyruvate (Sartorius). The resulting cell suspension was sieved through a 40-µm cell strainer to remove debris and stopped reactions with 10% FBS complete RPMI medium. After centrifugation at 800 × *g* for 5 min at room temperature, the cell pellet was resuspended in 7 mL of 30% Percoll (#17089101; GE Healthcare, Little Chalfont, UK) layered on top of 4 mL of 70% Percoll. The gradient was centrifuged at 800 × *g* for 20 min at room temperature without applying the brake. The cells were collected from the interphase between the 30% and 70% Percoll layers, washed with DPBS, and resuspended in complete RPMI medium on ice for subsequent flow cytometric analysis.

### Immunohistochemistry and Luxol Fast Blue staining

Mice were perfused with 40 mL of ice-cold 1X DPBS, followed by 40 mL of ice-cold 4% paraformaldehyde (PFA; Alfa Aesar, Lancashire, UK). Spinal cord tissues were collected and fixed in 2% PFA overnight at 4 °C. Subsequently, the tissues were embedded in paraffin for sectioning. The tissue sections were stained using hematoxylin and eosin (H&E) for general histological assessment and Luxol Fast Blue to evaluate myelin integrity.

Serial 4 µm paraffin-embedded tissue sections were deparaffinized using EZ Prep solution (Ventana Medical Systems, Inc., Tucson, AZ). The slides were incubated with anti-CD3 antibody (#790–4341; clone, 2GV6; Ventana Medical Systems, Tucson, Arizona), anti-CD68 antibody (#ab125212; polyclonal; Abcam, Cambridge, UK), anti-IFNγ antibody (#BS-0480R; polyclonal, Bioss, Boston, USA), and anti-IL-17A antibody (#BS-1183R; polyclonal, Bioss, Boston, USA) at a 1:100 dilution for 120 min using the automated Ventana Benchmark XT system (Ventana Medical Systems). Immunolabeling was visualized using Ultraview DAB Detection Kit (Ventana Medical Systems) according to the manufacturer’s protocol. All sections were counterstained with hematoxylin provided by Ventana reagents. The tissues were imaged using TissueFAXS microscopy (TissueGnostics, Vienna, Austria). Image projection and analysis were conducted with TissueFAXS software (TissueGnostics, Vienna, Austria), Fuji Image J (National Institutes of Health, USA), and QuPath version 0.6.0 [8].

### Isolation of splenocytes and lymph node cells

To prepare a single-cell suspension from spleen tissues, the spleen was pressed through a 40-µm strainer (Falcon, Glendale, AZ) using the plunger of a 5-mL syringe. The resulting cell suspension was centrifuged at 800 × *g* for 3 min at room temperature. Red blood cells were lysed by incubating the suspension in 10 mL of 1X red blood cell (RBC) lysis buffer (0.15 M ammonium chloride, 10 mM potassium bicarbonate, and 0.1 mM EDTA disodium salt dihydrate; pH 7.2) for 5 min at room temperature. Following this, the suspension was centrifuged at 400 × *g* for 3 min at room temperature. To remove debris, the cells were resuspended in 10 mL of 1X DPBS and gently pipetted up and down using a 10-ml serological pipette (SPL Life Sciences, Korea). After centrifugation at 400 × *g* for 3 min at room temperature, dead cells were removed using the Dead Cell Removal Kit (#130–090-101; MACS, Bergisch Gladbach, Germany) in conjunction with MS separation columns (#130–042-201; MACS).

For lymph node cell isolation, draining lymph nodes were collected and pooled from the brachial, axillary, and inguinal lymph regions. The lymph nodes were then mechanically dissociated by pressing them through a 70-µm strainer (Falcon) using the plunger of a 3-mL syringe. The resulting cell suspension was centrifuged at 400 × *g* for 3 min at room temperature. The pellet was then incubated in 5 mL of 1X RBC lysis buffer for 5 min at room temperature to lyse red blood cells. After a second centrifugation at 400 × *g* for 3 min, the pellet was resuspended in 5 mL of 1X DPBS and gently pipetted up and down with a 5-ml serological pipette (SPL Life Sciences). Following a final centrifugation at 400 × *g* for 3 min at room temperature, cells were resuspended in 5 mL of complete RPMI medium and stored on ice until further use.

### Flow cytometry: staining and analysis

Single-cell suspensions (2 × 10^6^) were washed twice with 1X DPBS and stained with either Fixable Viability Stain 780 (#565388; BD Biosciences; dilution 1:1000) or Zombie NIR (#423106; Biolegend; dilution 1:1000) for 10 min on ice. After centrifugation at 400 × *g* for 3 min, the cells were washed twice with flow cytometry buffer (1X DPBS containing 2% FBS and 0.05% sodium azide). Fc receptors were blocked by incubating the cells with purified anti-mouse CD16/32 antibodies (FcγRIII; #101302; Biolegend; dilution 1:100) for 5 min on ice. The cells were then stained with fluorophore-conjugated antibodies for 10 min on ice. Following two additional washes with flow cytometry buffer, the cells were fixed with 2% PFA at 4 °C for 20 min. After fixation, the cells were washed twice with 1X DPBS and stored in 1X DPBS containing 2 mM EDTA at 4 °C. For MOG_35–55_ TCR-independent intracellular cytokine staining, splenocytes and lymph node cells (2 × 10^6^) were incubated with 50 ng/mL phorbol-12-myristate-13-acetate (#P8139; Sigma-Aldrich, St. Louis, MO), 500 ng/mL ionomycin (#I0634; Sigma-Aldrich), and 5 µg/mL brefeldin A (BFA, Biolegend) for 4 h at 37 °C. After surface staining, the cells were fixed and permeabilized using the Cytofix/Cytoperm Fixation/Permeabilization Solution Kit (#554714; BD Biosciences) and incubated with intracellular antibodies overnight at 4 °C. The cells were then washed with 1 × Cytoperm solution and stored at 4 °C in 1X DPBS containing 2 mM EDTA. Flow cytometry data were acquired using Cytek Aurora, Cytek Northern Lights, BD LSR II and BD FACSLyric flow cytometry systems. Data analysis was performed using FlowJo software (BD Biosciences).

### Proliferation Assay

CD4^+^ T cell from 2D2 TCR mice (006912; The Jackson Laboratory) were purified from splenocytes (10 × 10^6^) and labeled with 5 µM carboxyfluorescein succinimidyl ester (CFSE; #65–0850-84, eBioscience, San Diego, CA) in 1X DPBS at room temperature for 10 min in the dark. The reaction was quenched by adding 5 mL of ice-cold complete RPMI medium, followed by a 5-min incubation at 4 °C. Afterward, the cells were centrifuged at 400 × *g* for 5 min and immediately washed twice with 5 mL of ice-cold complete RPMI. CD11b^+^ cells were purified from the splenocytes of mice post-EAE induction. CFSE labeled CD4^+^ T cells from 2D2 TCR were co-cultured with CD11b^+^ cells in 48-well plates for 3 days. After the co-culture period, the cells were stained with surface markers, fixed, and stored at 4 °C in 1X DPBS containing 2 mM EDTA, followed by flow cytometric analysis.

### Spinal cord tissue labeling and imaging

Spinal cord tissue labeling and imaging were conducted as described previously [14]. Briefly, mouse blood vessels were labeled by vessel painting via cardiac perfusion with Wheat Germ Agglutinin-Alexa Fluor 488 conjugate (30 mg/g body weight; #W11261, Invitrogen, Carlsbad, CA), followed by 4% PFA perfusion fixation. Afterward, the spinal cord was harvested, and vibratome Sects. (350 mm) of the tissue were post-fixed in 4% PFA at 15 °C for 30 min [51, 70]. The spinal cord was washed with 1X DPBS and storage in 0.1% PFA at 4 °C. The fixed tissues were then immersed in 2% Triton X-100 (Sigma-Aldrich) solution overnight at 15 °C for permeabilization. The spinal cord was then rinsed in 1X DPBS, followed by blocking with a buffer containing 2% Triton X-100, 10% normal goat serum, and 0.02% sodium azide in 1X DPBS for 2 h at 15 °C. Two primary antibodies were used to immuno-label the spinal cord: rabbit anti-lymphatic endothelial hyaluronan receptor 1 (Lyve-1; lymphatic endothelial marker; #ab14917, Abcam) and Alexa Fluor 647-conjugated anti-mouse CD3 antibody (#100209; clone, 17A2; Biolegend). The spinal cord tissues were incubated with the primary antibody, diluted in the dilution buffer (1:100, 0.25% Triton X-100, 1% normal goat serum, and 0.02% sodium azide in 1XDPBS) for 2 days at 15 °C to label CD3^+^ T cells and cellular structures in the spinal cord. Alexa Fluor 546-conjugated goat anti-rabbit IgG secondary antibody (#A11010; Thermo, Waltham, MA, USA; dilution, 1:200) was used to visualize the immunostained structures. Nuclei were counterstained with 4′,6-diamidino-2-phenylindole (DAPI; #D9542; Sigma-Aldrich). The labeled spinal cord tissues were then immersed overnight in the optical-clearing solution with a high refractive index (RapiClear 1.52 solution, SunJin Lab, Hsinchu, Taiwan), followed by an additional day of immersion in fresh RapiClear solution. The tissues were imaged using transmitted light and confocal microscopy (LSM800; Carl Zeiss, Jena, Germany). Image projection and analysis were conducted with LSM Image Browser (Carl Zeiss) and ZEN 3.2 software (Carl Zeiss).

### Immunoblotting

Murine microglial cells were purified using Percoll gradient as described previously and washed twice with 1X DPBS. The cells were resuspended in ice-cold radioimmunoprecipitation assay buffer (RIPA) buffer (50 mM Tris–HCl, 150 mM NaCl, 1% SDS, 1% sodium deoxycholate, and 1% Triton X-100; pH 7.5) containing protease inhibitors (2 µg/ml Aprotinin, 1 mg/ml Benzamidine, 1 µg/ml Pepstatin A, and 2 µg/ml Leupeptins; Sigma-Aldrich) and were sonicated with three 30 s bursts, separated by 1-min intervals, followed by incubation on ice for 30 min. After centrifugation at 14,000 × *g* for 10 min at 4 °C, the supernatant was transferred into a new eppendorf tube. SDS protein sample buffer (5X; 0.25 M Tris–HCl, 4% SDS, 16% 2-mercaptoethanol, 30% glycerol, and 0.002% bromophenol blue, pH 6.8) was added, and the mixture was incubated at 98 °C for 15 min. Protein samples were stored at −20 °C until further use in immunoblotting. Protein samples were separated by 12% SDS-PAGE in 1X running buffer (25 mM Tris–HCl, 190 mM glycine, and 0.1% SDS, pH 8.3) at 40–80 V. The separated proteins were transferred onto a polyvinylidene fluoride (PVDF) membrane (Merck Millipore, Billerica, MA) using 1X transfer buffer (25 mM Tris–HCl, 190 mM glycine, and 20% Methanol, pH 8.3) at 100 V for 2.5 h. The membrane was blocked with 10% non-fat milk in 1X TBST (20 mM Tris–HCl, 150 mM NaCl, and 0.1% Tween-20, pH 7.5) for 1 h and washed with 1X TBST four times for 15 min each. The membranes were incubated overnight at 4 °C with primary antibodies. After four additional washes with 1X TBST (15 min each), the membranes were incubated with HRP-conjugated secondary antibodies diluted in 10% milk for 2 h at room temperature. Following four final washes with 1X TBST (15 min each), signals were detected using an enhanced chemiluminescence (ECL) Western blotting substrate.

### Statistical analysis

GraphPad Prism 8.0 software was used for data analysis. Results are presented as mean ± SD. Statistical significance was determined using the Wilcoxon matched-pairs signed-rank test for EAE clinical scores and body weight changes. For two-group comparisons, statistical significance was assessed using the unpaired, two-tailed Student’s *t*-test. *P* values < 0.05 were considered statistically significant.

## Results

### Systemic depletion of ZNRF1 in mice promotes EAE progression and neuroinflammation

**Fig. 1:**
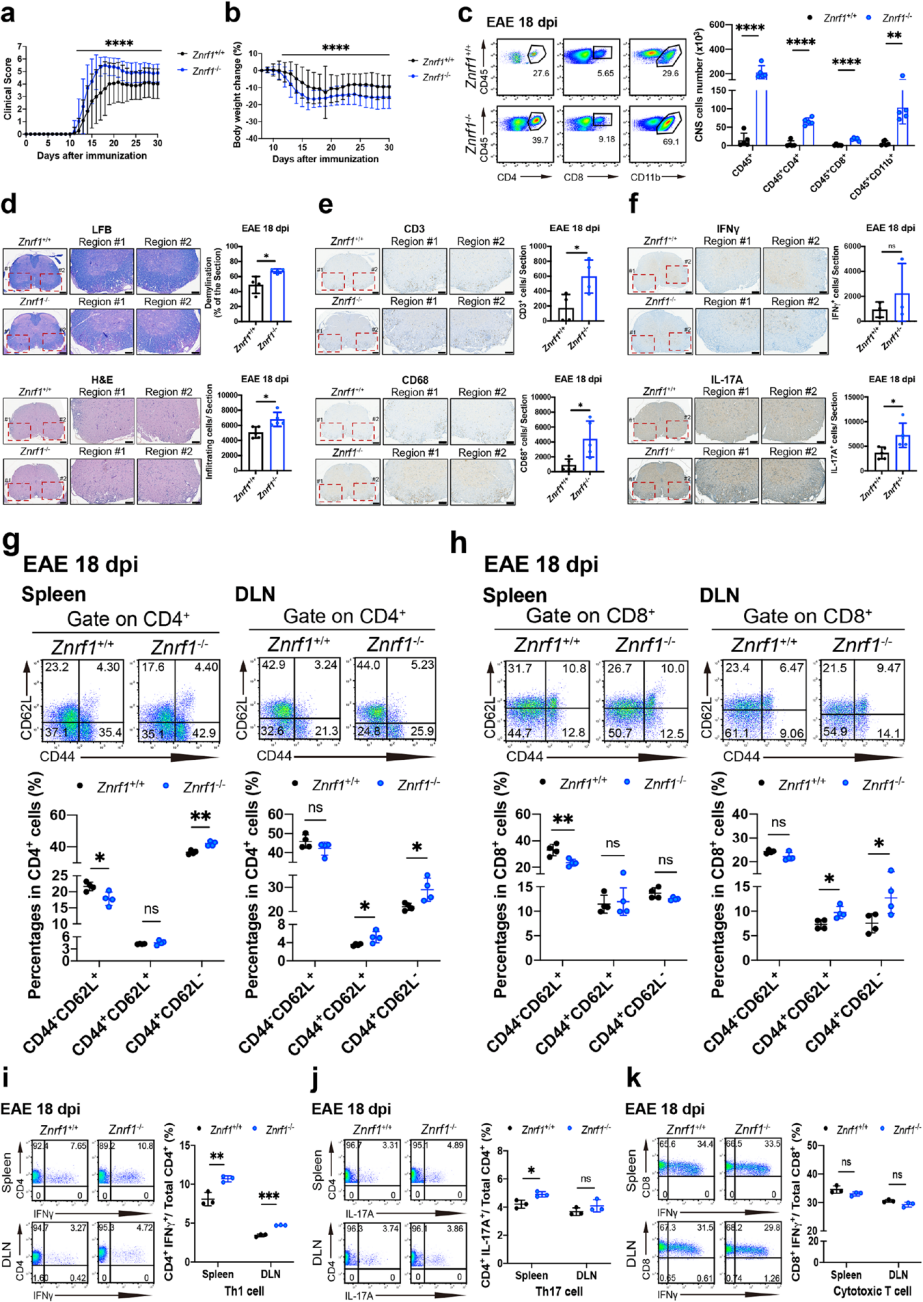
Systemic deletion of ZNRF1 promotes EAE pathogenesis and neuroinflammation in mice. Age-matched female *Znrf1*^+/+^ and *Znrf1*^−/−^ mice were immunized with 200 μg of MOG_35-55_ peptide emulsified in Complete Freund’s Adjuvant (CFA), followed by intraperitoneal (i.p.) injection of 200 ng pertussis toxin (PTX) to induce EAE. **a** Clinical scores of EAE progression were monitored and recorded daily (*N* = 40). **b** Body weights of immunized mice were measured and normalized to their respective weights at day 8 post-immunization (*N* = 40). **c** At day 18 post-EAE induction, spinal cords from female *Znrf1*^+/+^ and *Znrf1*^−/−^ mice were harvested to prepare single-cell suspensions. Cells were stained with antibodies against CD45, CD4, CD8, and CD11b, followed by flow cytometry analysis to quantify leucocytes (CD45^+^), T helper (Th) cells (CD45^+^CD4^+^), cytotoxic T cells (CD45^+^CD8^+^), and myeloid cells (CD45^+^CD11b^+^) (*N* = 6). Representative flow plots and quantified cell numbers of infiltrating immune cells in the CNS are presented. **d**-**f** Spinal cord tissue sections from *Znrf1*^+/+^ and *Znrf1*^−/−^ mice were collected and analyzed at day 18 post-EAE induction. **d** Tissue sections were stained with hematoxylin and eosin (H&E) to assess inflammatory cell infiltration and with luxol fast blue (LFB) to evaluate white matter demyelination. **e** and **f** Tissue sections were subjected to immunohistochemistry (IHC) using antibodies against CD3 (T cells) and CD68 (macrophages) (**e**) or IFNγ (Th1 cells) and IL-17A (Th17 cells) (**f**). **d**-**f** Scale bars: 200 μm (whole spinal cord sections) and 100 μm (enlarged region #1 and #2). **g** Flow cytometric analysis of naïve CD4^+^ T cells (CD4^+^CD44^−^CD62L^+^), central memory CD4^+^ T (T_CM_) cells (CD4^+^CD44^+^CD62L^+^), and effector memory CD4^+^ T (T_EM_) cells (CD4^+^CD44^+^CD62L^−^) in the spleens and draining lymph nodes (DLNs) of *Znrf1*^+/+^ (N = 4) and *Znrf1*^−/−^ mice (*N* = 4) at day 18 post-EAE induction. **h** Flow cytometric analysis of naïve CD8^+^ T cells (CD8^+^CD44^−^CD62L^+^), CD8^+^ T (T_CM_) cells (CD8^+^CD44^+^CD62L^+^), and effector memory CD8^+^ T (T_EM_) cells (CD8^+^CD44^+^CD62L^−^) in the spleens and DLNs of *Znrf1*^+/+^ (*n* = 4) and *Znrf1*^−/−^ mice (*N* = 4) at day 18 post-EAE induction. **i-k** Flow cytometric analysis of Th1 (CD4^+^IFNγ^+^) cells (*i*), Th17 (CD4^+^IL-17A^+^) cells (**j**), and cytotoxic T (CD8^+^IFNγ^+^) cells (**k**) in the spleens and DLNs of *Znrf1*^+/+^ (*n* = 3) and *Znrf1*^−/−^ mice (*N* = 3) at day 18 post-EAE induction. (**a** and **b**) Data are presented as mean ± SD. ns, not significant. *P < 0.05, **P < 0.01, ***P < 0.001, ****P < 0.0001, determined by the Wilcoxon matched-pairs signed-rank test. **d-f** Representative spinal cord sections and quantification of infiltrating immune cells or demyelination in *Znrf1*^+/+^ and *Znrf1*^−/−^ mice are shown. **g**-**k** Representative flow cytometry plots and quantified T cell populations in the spleens and DLNs. **c**-**k** Data are presented as mean ± SD. ns, not significant. *P < 0.05, **P < 0.01, ***P < 0.001, ****P < 0.0001, determined by the unpaired Student’s *t*-test

ZNRF1, originally identified as a regulator during nerve injury, is known to participate in oxidative stress-induced neuronal death and axonal degeneration [3, 85, 86]. However, its role in neuroinflammation remains unclear. To address this, we explored the involvement of ZNRF1 in the pathogenesis of experimental autoimmune encephalomyelitis (EAE), a widely used mouse model that recapitulates certain aspects of the inflammatory cascade in human MS [18]. Wild-type (*Znrf1*^+/+^) and *Znrf1*^−/−^ mice were immunized with MOG_35-55_ to induce EAE. Compared to wild-type controls, *Znrf1*^−/−^ mice exhibited significantly higher clinical scores after EAE induction (Fig. 1a). Additionally, the body weights in *Znrf1*^−/−^ mice were changed, inversely correlating with enhanced clinical severity of EAE (Fig. 1b). This was corroborated by flow cytometric analysis, which revealed significantly increased numbers of infiltrating leucocytes (CD45^+^), CD4^+^ T cells (CD45^+^CD4^+^), cytotoxic T cells (CD45^+^CD8^+^), and myeloid cells (CD45^+^CD11b^+^) in the spinal cords of *Znrf1*^−/−^ mice at both day 18 (Fig. 1c) and day 30 (Fig. S3b) following EAE induction. At the peak (day 18) and late stages (day 30) of the disease, spinal cords were harvested and analyzed using hematoxylin and eosin (H&E) and Luxol fast blue (LFB) staining. H&E staining showed significantly greater immune cell infiltration in the spinal cords of *Znrf1*^−/−^ mice (Fig. 1d and Fig. S3c). Consistently, LFB staining also revealed increased demyelination in the spinal cord of *Znrf1*^−/−^ mice compared to *Znrf1*^+/+^ controls (Fig. 1d and Fig. S3c). Immunohistochemistry (IHC) analysis further confirmed elevated levels of infiltrating CD3^+^ T cells and macrophages (CD68^+^) in the spinal cords of *Znrf1*^−/−^ mice at both day 18 and day 30 post-EAE induction (Fig. 1e and Fig. S3c). In addition, IHC revealed increased infiltration of Th17 cells (IL-17A^+^) and a trend toward higher numbers of Th1 cells (IFNγ^+^) in the spinal cords of *Znrf1*^−/−^ mice at the same time points (Fig. 1f and Fig. S3c). Together, these data demonstrate that systemic ZNRF1 deficiency exacerbates EAE progression and neuroinflammation.

### ZNRF1 deficiency enhances T cell activation and differentiation in peripheral lymphoid organs

Given the central role of CD4^+^ T cells in MS pathogenesis [5, 16, 45], we evaluated whether ZNRF1 influences T cell development and activation by analyzing T cell populations in the spleen and thymus of *Znrf1*^+/+^ and *Znrf1*^−/−^ mice. T cell development appeared unaffected by ZNRF1 deficiency, as no differences were observed in the frequencies of T cell subsets in either the spleen or thymus (Fig. S1a -S1f). Upon TCR stimulation, *Znrf1*^−/−^ T cells exhibited comparable proliferation to *Znrf1*^+/+^ T cells (Fig. S2a), and no differences were observed in IL-2 or IFNγ production between the two genotypes (Fig. S2b). Furthermore, activation markers, including CD25 and CD69, on CD4^+^ T cells were similar between *Znrf1*^+/+^ and *Znrf1*^−/−^ mice (Fig. S2d-e), and the frequency of Th1 cells (CD4^+^IFNγ^+^) remained unchanged (Fig. S2c). Similarly, central memory T cell markers CD44 and CD62L on CD4^+^ T cells did not differ between *Znrf1*^+/+^ and *Znrf1*^−/−^ mice (Fig. S2f). We also assessed CD4^+^ T cell polarization toward Th1 and Th17 lineages and found that ZNRF1 did not alter either pathway (Fig. S2g and S2h). These findings suggest that ZNRF1 is dispensable for T cell development, activation, and Th1/17 polarization under both homeostatic and stimulatory conditions.

We then investigated how ZNRF1 suppresses EAE induction by analyzing T cell activation in peripheral lymphoid organs, including the spleen and draining lymph nodes (DLNs), at the peak and chronic stages of the disease. Compared to *Znrf1*^+/+^ mice, naïve CD4^+^ T (CD44^−^CD62L^+^) cells were decreased, while effectors memory CD4^+^ T (CD44^+^CD62L^−^) cells in both spleens and DLNs of *Znrf1*^−/−^ mice were significantly increased at day 18 after EAE induction (Fig. 1g). Similarly, at day 30 post-immunization, naïve CD4^+^ T cells were decreased, whereas central memory CD4^+^ T (T_CM_) cells and effectors memory CD4^+^ T (T_EM_) cells were also elevated in peripheral lymphoid organs of *Znrf1*^−/−^ mice (Fig. S3e). In addition, naïve CD8^+^ T (CD8^+^CD44^−^CD62L^+^) cells in the spleen of *Znrf1*^−/−^ mice, compared to *Znrf1*^+/+^ mice, were significantly decreased at both day 18 (Fig. 1h) and day 30 (Fig. S3f) post-EAE induction. Conversely, CD8^+^ T_CM_ cells (CD8^+^CD44^+^CD62L^+^) and CD8^+^ T_EM_ cells (CD8^+^CD44^+^CD62L^−^) were markedly increased in the DLNs of *Znrf1*^−/−^ mice at both time points after EAE induction (Fig. 1h and S3f). These data suggest that ZNRF1 deficiency enhances T cell activation and differentiation in peripheral lymphoid organs. We further examined the polarization of effector T cell subsets-Th1 (CD4^+^IFNγ^+^), Th17 (CD4^+^IL-17A^+^), and cytotoxic CD8^+^ T (CD8^+^IFNγ^+^) cells in *Znrf1*^+/+^ and *Znrf1*^−/−^ mice in both spleens and DLNs during EAE progression. At baseline, the frequencies of Th1, Th17, and cytotoxic CD8^+^ T cells were comparable between naïve *Znrf1*^+/+^ and *Znrf1*^−/−^ mice (Fig. S2i-S2k). Following EAE induction, Th1 cells were significantly increased in both spleens and DLNs of *Znrf1*^−/−^ mice compared to *Znrf1*^+/+^ controls at both day 18 and day 30 (Fig. 1i and S3h). Th17 cells were significantly increased in DLNs of *Znrf1*^−/−^ mice at day 30, and slightly elevated in *Znrf1*^−/−^ spleen at day 18 after immunization (Fig. 1j and S3i). Furthermore, the cytotoxic CD8^+^ T cell population was significantly increased only in the spleens of *Znrf1*^−/−^ mice at day 30 after EAE (Fig. 1k and S3j). These data suggest that ZNRF1 deficiency promotes effector T cell activation, differentiation, and polarization during EAE.

### ZNRF1 deficiency in microglia does not affect EAE pathogenesis

**Fig. 2:**
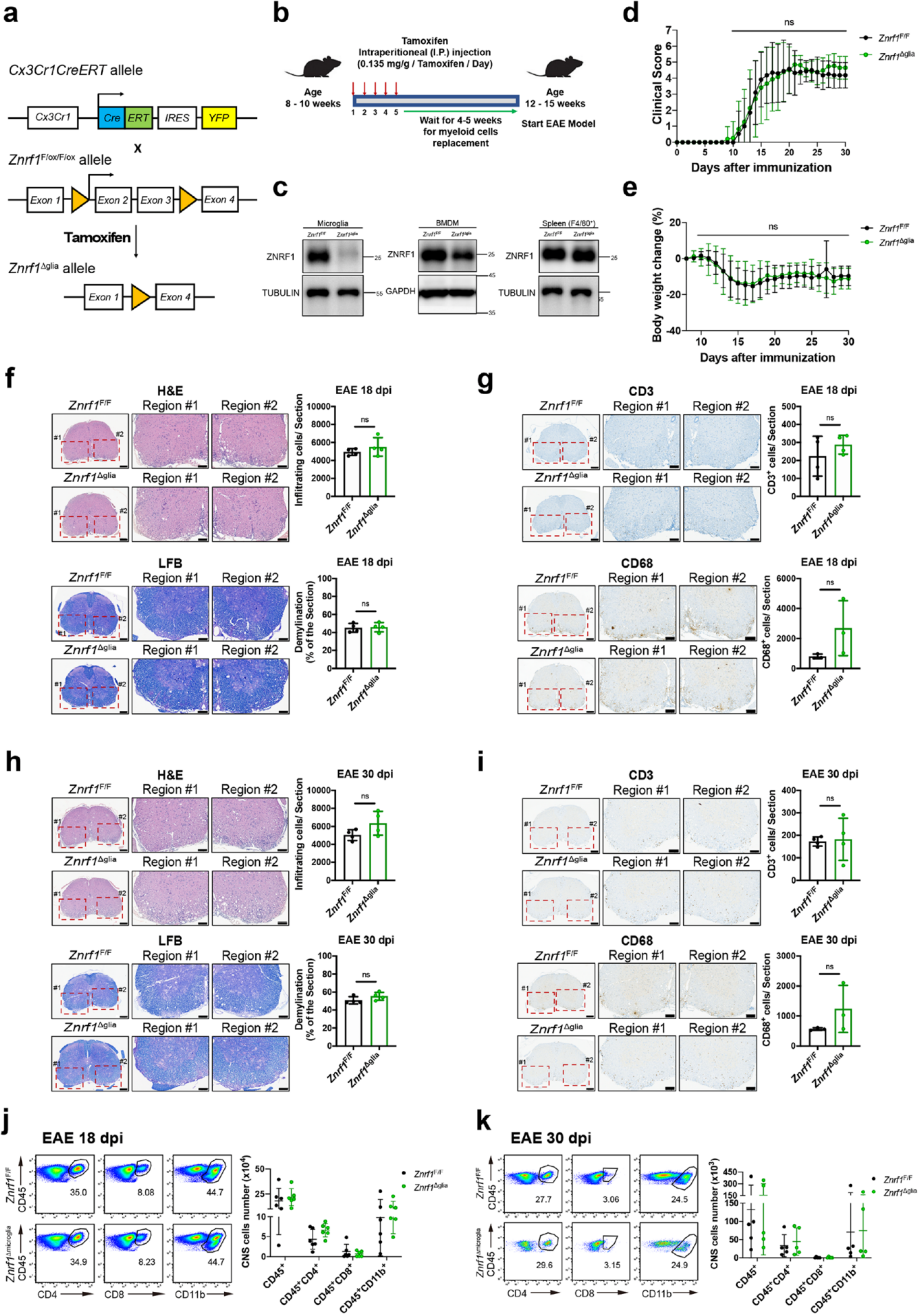
ZNRF1 deficiency in microglia does not affect EAE pathogenesis and neuroinflammation in mice. **a** Schematic diagram illustrating the tamoxifen-inducible *Cx3cr1-Cre* (*Cx3cr1-CreERT*) allele, the *Znrf1*^Flox^ allele, and the deleted *Znrf1* allele (*Znrf1*^Δglia^) after *Cre*-mediated recombination driven by tamoxifen (TAM) administration. ERT, estrogen receptor; IRES, internal ribosome entry site; YFP, yellow fluorescent protein. **b** Schematic diagram depicting the generation of *Znrf1*^Δglia^ mice. 5-week-old *Znrf1*^Flox/Flox^:*Cx3cr1-CreERT* mice were i.p. injected with TAM (0.135 mg/g body weight) daily for five days, followed by a 4-week waiting period to allow the replacement of myeloid cells. **c** Microglia, BMDMs, and splenic F4/80^+^ cells were isolated from *Znrf1*^F/F^ and *Znrf1*^Δglia^ mice at 4–5 weeks post-TAM administration and subjected to immunoblotting analysis for ZNRF1 expression. (**d**-**i**) Age-matched female *Znrf1*^F/F^ and *Znrf1*^Δglia^ mice were immunized with 200 μg of MOG_35-55_ peptide emulsified in CFA, followed by i.p. injection of 200 ng PTX to induce EAE. Clinical scores of EAE progression were monitored and recorded daily (*N* = 15) (**d**). Body weights of immunized mice were measured and normalized to their respective weights at day 8 post-immunization (**e**). Spinal cords from *Znrf1*^F/F^ and *Znrf1*^Δglia^ mice were collected at day 18 (**f** and **g**) and day 30 (**h** and **i**) after EAE induction. (**f** and **h**) Tissue sections were subjected to H&E and LFB staining. (**g** and **i**) Tissue sections were subjected to IHC using antibodies against CD3 (T cells) and CD68 (macrophages). (**f**-**i**) Scale bars: 200 μm (whole spinal cord sections) and 100 μm (enlarged region #1 and #2). (**j**-**k**) Single-cell suspensions were prepared from spinal cords of female *Znrf1*^F/F^ and *Znrf1*^Δglia^ mice at day 18 (**j**) and day 30 (**k**) post-EAE induction, followed by flow cytometry analysis of leucocytes (CD45^+^), Th cells (CD45^+^CD4^+^), cytotoxic T cells (CD45^+^CD8^+^), and myeloid cells (CD45^+^CD11b^+^) (*N* = 6). Representative flow cytometry plots and quantified numbers of infiltrating immune cells in the CNS are presented. (**d** and **e**) Data are presented as mean ± SD. ns, not significant. Statistical significance was determined by the Wilcoxon matched-pairs signed-rank test. (**f**-**i**) Representative spinal cord sections and quantification of infiltrating immune cells or demyelination in *Znrf1*^F/F^ and *Znrf1*^Δglia^ mice are shown. (**d**-**k**) Data are presented as mean ± SD. ns, not significant. Statistical significance was determined by the unpaired Student’s *t*-test

Besides T cells, myeloid cells, including macrophages and DCs, as well as microglia (the resident macrophages in the CNS), contribute to the pathogenesis of EAE [21]. To investigate whether microglial ZNRF1 influences EAE, we crossed *Znrf1*^F/F^ mice with *Cx3cr1-CreERT2* transgenic mice [27, 90] to generate *Znrf1*^F/F^: *Cx3cr1-CreERT2* mice (Fig. 2a). Microglial *Znrf1* was deleted by intraperitoneal tamoxifen injection, followed by a 30-day waiting period (referred to hereafter as *Znrf1*^Δglia^) (Fig. 2b). Given that the half-life of myeloid-derived macrophages is approximately four weeks, while that of microglia exceed six months [27, 72, 90], myeloid-derived macrophages were replaced by newly undeleted macrophages derived from bone marrow progenitors, whereas the long-lived *Znrf1*-deleted microglia persisted. To confirm the efficiency of *Znrf1* deletion in microglia, we assessed ZNRF1 protein levels in microglia isolated from wild-type and *Znrf1*^Δglia^ mice by immunoblotting, and observed a significant reduction in ZNRF1 protein levels in *Znrf1*-deleted microglia (Fig. 2c). By contrast, ZNRF1 protein levels in bone marrow-derived macrophages (BMDMs) and F4/80^+^ splenic macrophages were comparable between wild-type and *Znrf1*^Δglia^ mice (Fig. 2c). We then induced EAE in both control and *Znrf1*^Δglia^ mice. Upon EAE induction, *Znrf1*^F/F^ and *Znrf1*^Δglia^ mice exhibited similar clinical scores and body weight changes (Fig. 2d and e). Consistent with these observations, H&E staining revealed comparable immune cell infiltration in the spinal cords of *Znrf1*^F/F^ and *Znrf1*^Δglia^ mice at both day 18 and day 30 after EAE induction (Fig. 2f and h). LFB staining also showed no exacerbated demyelination in *Znrf1*^Δglia^ spinal cords (Fig. 2f and h). IHC analysis further demonstrated similar infiltration of CD3^+^ T cells and CD68^+^ macrophages in the spinal cords of both groups at both time points (Fig. 2g and i). Flow cytometric analysis confirmed that the numbers of infiltrating leucocytes, CD4^+^ T helper (Th) cells, cytotoxic T cells, and myeloid cells were comparable in the spinal cords of both groups (Fig. 2j and k). Together, these data indicate that microglia ZNRF1 does not significantly contribute to the pathogenesis of EAE.

### ZNRF1 deficiency in myeloid cells aggravates EAE

**Fig. 3:**
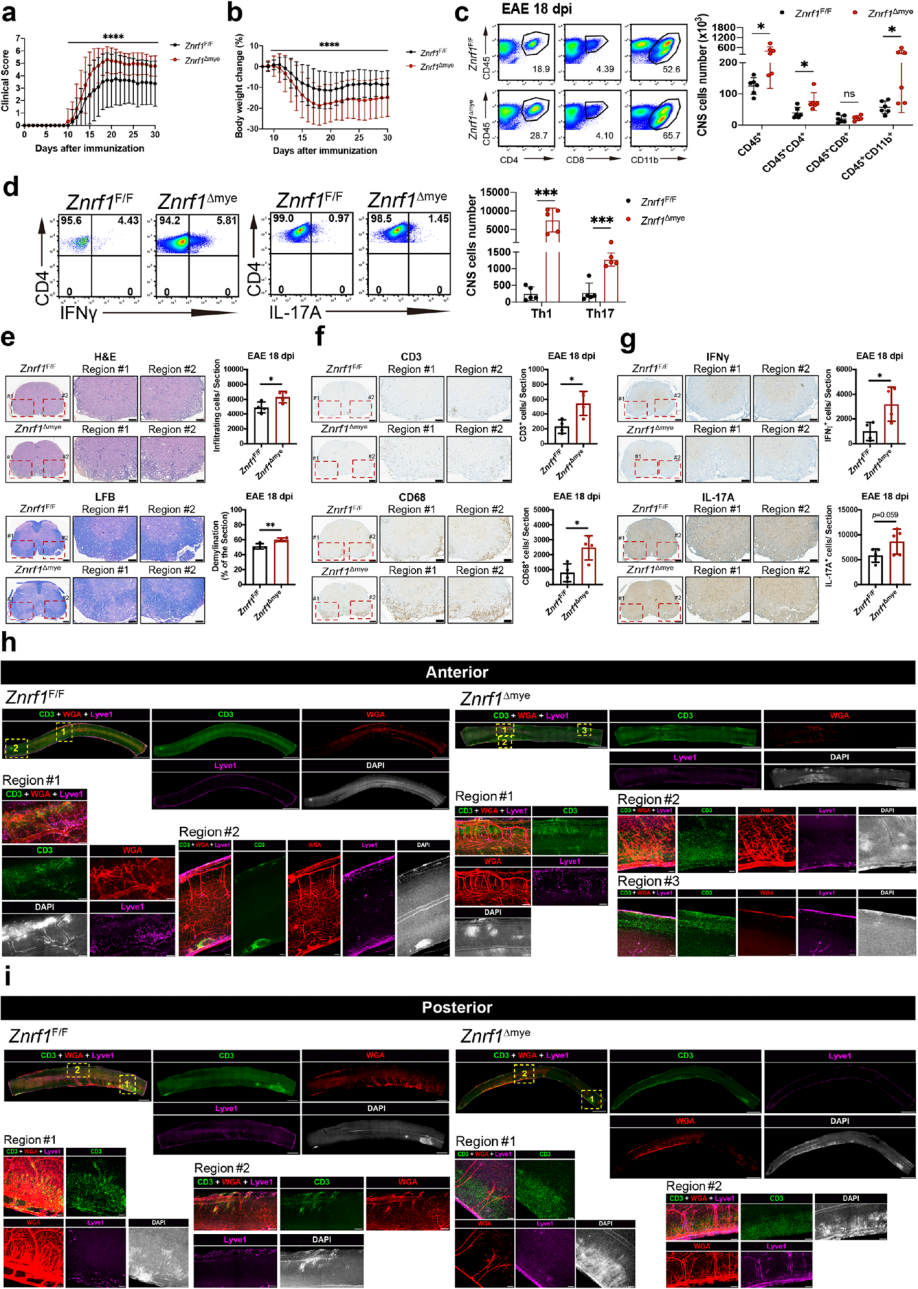
ZNRF1 deficiency in myeloid cells promotes EAE pathogenesis and neuroinflammation in mice. Age-matched female *Znrf1*^F/F^ and *Znrf1*^Δmye^ mice were immunized with 200 μg of MOG_35-55_ peptide emulsified in CFA, followed by i.p. injection of 200 ng PTX to induce EAE. **a** Clinical scores of EAE progression were monitored and recorded daily (N = 42). **b** Body weights of immunized mice were measured and normalized to their respective weights at day 8 post-immunization. **c** At day 18 post-EAE induction, spinal cords from female *Znrf1*^F/F^ and *Znrf1*^Δmye^ mice were harvested to prepare single-cell suspensions. Cells were stained with antibodies against CD45, CD4, CD8, and CD11b, followed by flow cytometry analysis to quantify leucocytes (CD45^+^), Th cells (CD45^+^CD4^+^), cytotoxic T cells (CD45^+^CD8^+^), and myeloid cells (CD45^+^CD11b^+^) (*N* = 6). **d** Flow cytometry analysis of Th1 (CD4^+^IFNγ^+^) and Th17 (CD4^+^IL-17A^+^) cells in the spinal cords of *Znrf1*^F/F^ (*N* = 5) and *Znrf1*^Δmye^ mice (*N* = 5) at day 18 post-EAE induction. **e**–**g** Spinal cord tissue sections from *Znrf1*^F/F^ and *Znrf1*^Δmye^ mice were collected and quantified at day 18 post-EAE induction. Tissue sections were subjected to H&E and LFB staining (**e**), and IHC staining using antibodies against CD3 (T cells) and CD68 (macrophages) (**f**) or IFNγ (Th1 cells) and IL-17A (Th1 cells) (**g**). Scale bars: 200 μm (whole spinal cord sections) and 100 μm (enlarged region #1 and #2). **h**-**i** Whole-mount imaging of the anterior (**h**) and posterior (**i**) spinal cord was performed in *Znrf1*^F/F^ and *Znrf1*^Δmye^ mice after EAE induction. At day 18 post-EAE induction, *Znrf1*^F/F^ and *Znrf1*^Δmye^ mice were perfused with wheat germ agglutinin (WGA) to label blood vessels (red). Spinal cords were collected and immunostained with antibodies against CD3 (green) to detect CD3^+^ T cells, lymphatic vessel endothelial hyaluronan receptor 1 (Lyve1) (magenta) for lymph vessels, and DAPI (white) for nuclei. Tissue clearing was performed as described in the METHODS section. Confocal images from whole-mount spinal cords and enlarged regions are shown. Scale bars: 2000 μm (whole-mount anterior spinal cord for *Znrf1*^F/F^ and *Znrf1*^Δmye^ mice and whole-mount posterior spinal cord for *Znrf1*^Δmye^ mice), 1000 μm (whole-mount posterior spinal cord for *Znrf1*^F/F^ mice), 200 μm (enlarged region #1), 100 μm (enlarged region #2 and #3). (**a**,**b**) Data are presented as mean ± SD. ns, not significant. *P < 0.05, **P < 0.01, ***P < 0.001, ****P < 0.0001, determined by the Wilcoxon matched-pairs signed-rank test. (**c** and **d**) Representative flow cytometry plots and quantified numbers of infiltrating immune cells in the CNS are shown. Data are presented as mean ± SD. ns, not significant. *P < 0.05, **P < 0.01, ***P < 0.001, determined by the unpaired Student’s t-test. (**e**–**g**) Representative spinal cord sections and quantification of infiltrating immune cells or demyelination in *Znrf1*^F/F^ and *Znrf1*^Δmye^ mice. Data are presented as mean ± SD. ns, not significant. *P < 0.05, **P < 0.01, determined by the unpaired Student’s t-test

To investigate the role of ZNRF1 in myeloid cells during EAE, we crossed *Znrf1*^F/F^ mice with *LysMCre* knock-in mice [15, 78] to generate myeloid cell-specific ZNRF1-deficient (hereafter referred to as *Znrf1*^Δmye^) mice and induced EAE in both control and *Znrf1*^Δmye^ mice. Unlike *Znrf1*^Δglia^ mice, *Znrf1*^Δmye^ mice developed markedly more severe EAE phenotypes, exhibiting elevated clinical scores and more pronounced changes in body weight (Fig. 3a and b). Flow cytometric analysis corroborated these results, showing significantly increased infiltrating leucocytes, CD4^+^ T cells, and myeloid cells, but not cytotoxic CD8^+^ T cells, in spinal cords of *Znrf1*^Δmye^ mice at both day 18 (Fig. 3c) and day 30 (Fig. S5a) after EAE induction. Notably, *Znrf1*^Δmye^ mice also exhibited significantly elevated Th1 and Th17 cells (Fig. 3d).

Consistent with these findings, H&E staining revealed increased immune cell infiltration in spinal cords of *Znrf1*^Δmye^ mice (Fig. 3e and S5b). In addition, LFB staining showed more extensive demyelination in *Znrf1*^Δmye^ spinal cords (Fig. 3e and S5b). IHC analysis further confirmed greater infiltration of CD3^+^ T cells and CD68^+^ macrophages in *Znrf1*^Δmye^ mice at both day 18 and day 30 post-induction (Fig. 3f and S5b). IHC analysis also demonstrated increased infiltration of Th1 cells (IFNγ^+^) in *Znrf1*^Δmye^ mice at both time points (Fig. 3g and S5b), while Th17 cells (IL-17A^+^) were significantly increased at day 30 and modestly elevated at day 18 post-induction (Fig. 3g and S5b). Inflammatory lesions in spinal cords typically develop randomly following EAE induction, with immune cells infiltrating within these lesions. To visualize the localization of infiltrating CD3^+^ T cells in the spinal cords, we performed whole-mount spinal cord immunostaining with anti-CD3 antibodies and subjected the samples to tissue clearing as described in the Methods. Consistent with the above findings, we observed increased CD3^+^ T cell infiltration in both anterior and posterior regions of the spinal cord in *Znrf1*^Δmye^ mice at day 18 post-EAE induction (Fig. 3h and i). Together, these data indicate that ZNRF1 in myeloid cells suppresses EAE progression and associated neuroinflammation.

### ZNRF1 deficiency in myeloid cells increases T cell priming in peripheral lymphoid organs after EAE induction

**Fig. 4:**
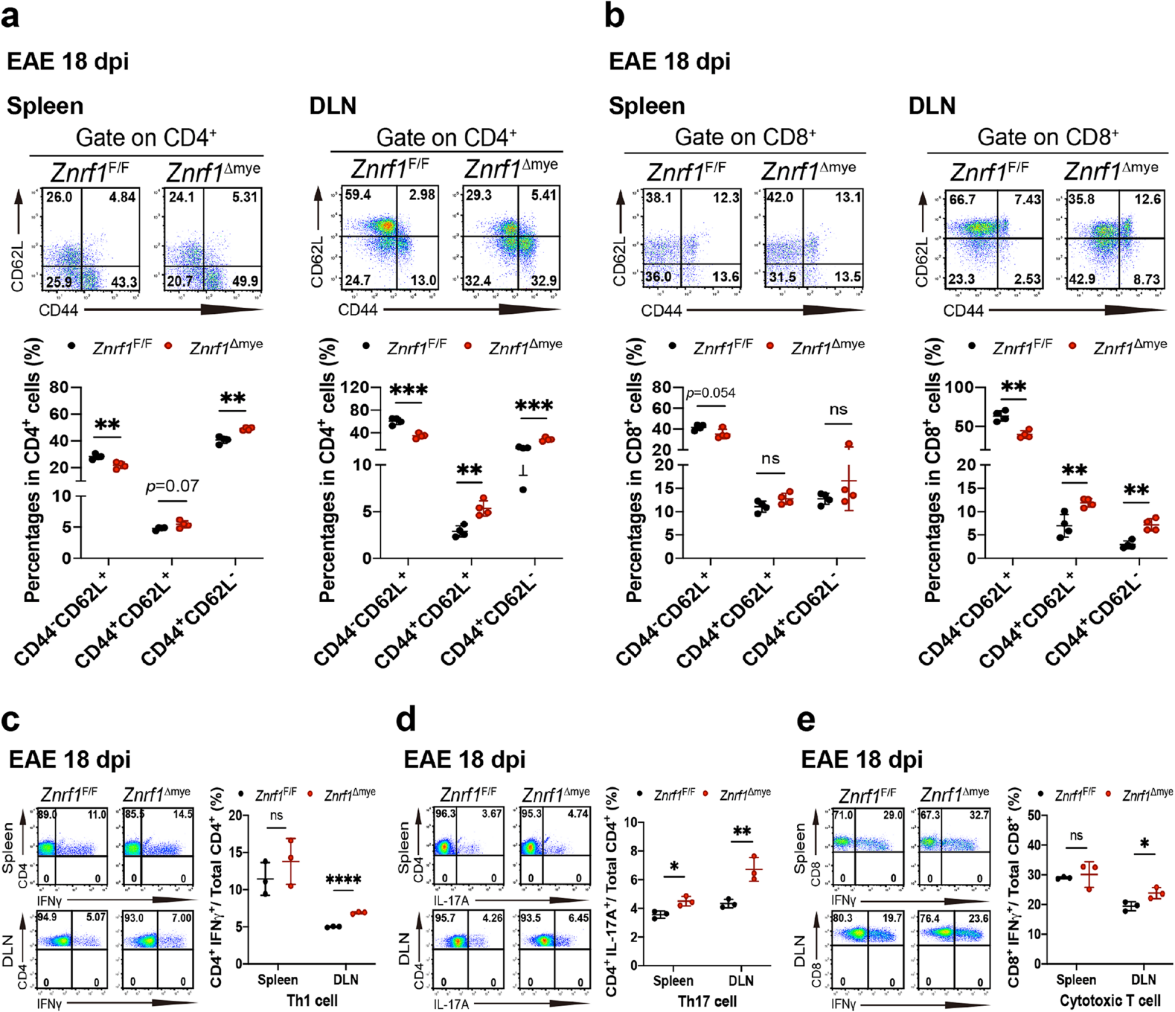
ZNRF1 deficiency in myeloid cells enhances T cell differentiation in peripheral lymphoid organs following EAE induction. **a** Flow cytometry analysis of naïve CD4^+^ T cells (CD4⁺CD44⁻CD62L⁺), CD4^+^ T_CM_ cells (CD4⁺CD44^+^CD62L⁺), and CD4^+^ T_EM_ cells (CD4⁺CD44^+^CD62L^−^) in the spleens and DLNs of *Znrf1*^F/F^ (N = 4) and *Znrf1*^Δmye^ mice (*N* = 4) at day 18 post-EAE induction. **b** Flow cytometry analysis of naïve CD8^+^ T cells (CD8⁺CD44⁻CD62L⁺), CD8^+^ T_CM_ cells (CD8⁺CD44^+^CD62L⁺), and effector memory CD8^+^ T_EM_ cells (CD8⁺CD44^+^CD62L^−^) in the spleens and DLNs of *Znrf1*^F/F^ (*N* = 4) and *Znrf1*^Δmye^ mice (*N* = 4) at day 18 post-EAE induction. (**c**-**e**) Flow cytometry analysis of Th1 cells (CD4⁺IFNγ⁺) (**c**), Th17 cells (CD4⁺IL-17A⁺) (**d**), and cytotoxic T cells (CD8^+^IFNγ^+^) (**e**) in the spleens and DLNs of *Znrf1*^F/F^ (*N* = 3) and *Znrf1*^Δmye^ mice (*N* = 3) at day 18 post-EAE induction. Representative flow cytometry plots and quantified numbers of T cell populations in the spleen and DLNs are shown. Data are displayed as mean ± SD. ns, not significant. *P < 0.05, **P < 0.01, ***P < 0.001, ****P < 0.0001, determined by the unpaired Student’s *t*-test

To further investigate how ZNRF1 deficiency in myeloid cells enhances EAE progression, we analyzed T cell priming in peripheral lymphoid tissues (spleen and DLNs) after EAE induction. During the early phase (day 9) of EAE induction, CD69 expression on CD4^+^ T cells was significantly increased in both spleens and DLNs of *Znrf1*^Δmye^ mice (Fig. S4a). At the same time point, Th1 and Th17 cell populations were also significantly elevated (Fig. S4d and S4e). We next examined various CD4^+^ T cell populations in these tissues. Our results revealed that naïve CD4^+^ T cells (CD44^−^CD62L^+^) were significantly reduced in both spleens and DLNs of *Znrf1*^Δmye^ mice at day 18 and 30 after EAE induction, whereas CD4^+^ T_CM_ cells remained comparable to control (Fig. 4a and S5c). In contrast, CD4^+^ T_EM_ cells were significantly increased in both spleens and DLNs of *Znrf1*^Δmye^ mice at day 18 after immunization (Fig. 4a). We also examined CD8^+^ T cell subsets. Compared to *Znrf1*^F/F^ mice, CD8^+^ T_CM_ cells were significantly increased in DLNs of *Znrf1*^Δmye^ mice at both day 18 and 30 (Fig. 4b and S5d). In addition, CD8^+^ T_EM_ cells were slightly increased in DLNs at day 18 and in spleens at day 30 in *Znrf1*^Δmye^ mice following EAE induction (Fig. 4b and S5d).

We further investigated the differentiation of effector T cell subsets (Th1, Th17, and cytotoxic CD8^+^ T cell) in both spleen and DLN of *Znrf1*^F/F^ and *Znrf1*^Δmye^ mice during EAE. Th1 cells were significantly increased in DLNs at day 18 and in spleens at day 30 post-EAE induction in *Znrf1*^Δmye^ mice (Fig. 4c and S5e). However, Th17 cells were elevated in both spleens and DLNs of *Znrf1*^Δmye^ mice at both day 18 and day 30 (Fig. 4d and S5f). Moreover, CD8^+^ cytotoxic T cells were significantly increased in the DLNs, but not the spleen, of *Znrf1*^Δmye^ mice at both time points (Fig. 4e and S5g). Taken together, these results suggest that ZNRF1 deficiency in myeloid cells promotes robust T cell activation and differentiation during EAE.

### ZNRF1 regulates antigen-specific T cell proliferation and activation after EAE induction

**Fig. 5:**
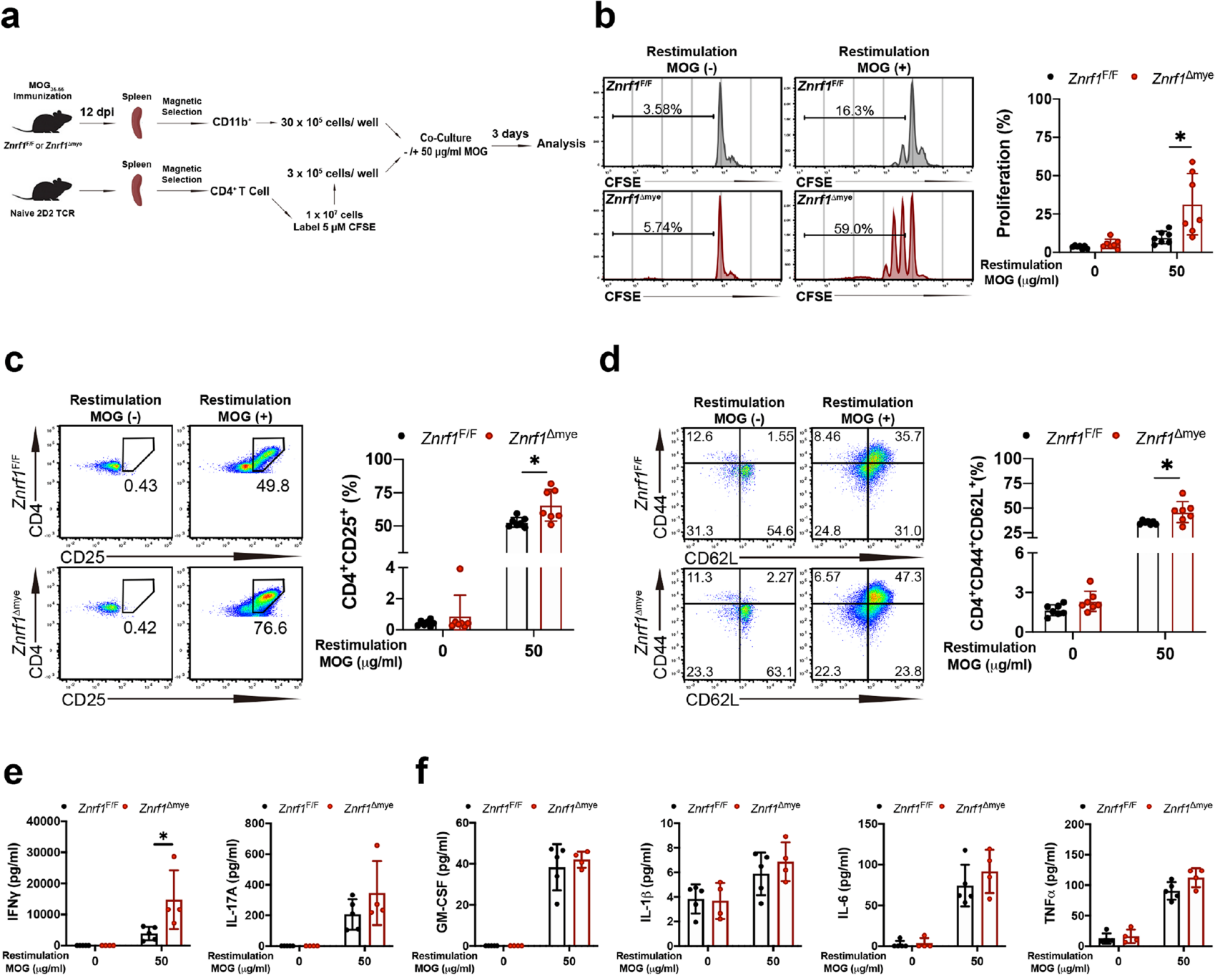
ZNRF1 deficiency in myeloid cells enhances T cell proliferation and activation in peripheral lymphoid organs after EAE induction. **a** Schematic diagram of the experimental setup. *Znrf1*^F/F^ and *Znrf1*^Δmye^ mice were immunized with MOG_35–55_ without PTX administration. On day 12 post-immunization, CD11b^+^ cells were isolated from the spleens of immunized *Znrf1*^F/F^ and *Znrf1*^Δmye^ mice using magnetic selection. CD4^+^ T cells were isolated from the spleens of naive 2D2 mice via magnetic selection and labeled with 5 μM carboxyfluorescein succinimidyl ester (CFSE). CD11b^+^ and CD4^+^ cells were co-cultured at a 10:1 ratio in the absence or presence of 50 μg/mL MOG_35–55_ for 3 days, followed by flow cytometry analysis. **b** Flow cytometric analysis of CD4^+^ T cell proliferation in co-cultures of CD11b^+^ cells from *Znrf1*^F/F^ (N = 7) and *Znrf1*^Δmye^ (*N* = 7) mice with naive 2D2 CD4^+^ T cells for 3 days, either in the absence or presence of 50 µg/ml MOG_35-55_. Representative flow plots (top) and quantification of the percentage of proliferating CD4^+^ T cells (bottom) are shown. **c** Flow cytometric analysis of activated T cells (CD4^+^CD25^+^) among total T cells in *Znrf1*^F/F^ (*N* = 7) and *Znrf1*^Δmye^ (N = 7) mice 3 days after co-culture. **d** Flow cytometric analysis of CD4^+^ T_CM_ cells (CD4^+^CD44^+^CD62L^+^) among total T cells in *Znrf1*^F/F^ (*N* = 7) and *Znrf1*^Δmye^ (*N* = 7) mice 3 days after co-culture. **e**–**f** Cytokine levels in supernatants from myeloid cells-T cell cocultures for 3 days were determined by multiplex immunoassay. All data are presented as mean ± SD. ns, not significant. *P < 0.05, **P < 0.01, determined by the unpaired Student’s *t*-test

Antigen-activated T cells are known to be primed by antigen-presenting cells (APCs) in peripheral lymphoid organs before migrating to the CNS [31, 58]. Based on the results shown above, we hypothesized that ZNRF1 in myeloid cells regulates APC function and thereby contributes to antigen-specific T cell responses during EAE. To test this hypothesis, we first co-cultured carboxyfluorescein succinimidyl ester (CFSE)-labeled CD4^+^ T cells from the spleens of 2D2 TCR transgenic mice with CD11b^+^ myeloid cells isolated from either un-immunized *Znrf1*^F/F^ or *Znrf1*^Δmye^ mice in the presence or absence of MOG_35-55_ for 3 days (Fig. S6a). MOG_35-55_ stimulation led to significantly increased proliferation of CD4^+^ T cells co-cultured with *Znrf1*^Δmye^ myeloid cells compared with *Znrf1*^F/F^ controls (Fig. S6d). We next repeated the co-culture using CFSE-labeled CD4^+^ T cells from 2D2 mice and CD11b^+^ myeloid cells isolated from immunized *Znrf1*^F/F^ and *Znrf1*^Δmye^ mice at day 12 post-EAE, again in the presence or absence of MOG_35-55_ for 3 days (Fig. 5a). Consistent with the earlier experiments, MOG_35-55_ stimulation induced significantly greater proliferation of CD4^+^ T cells co-cultured with *Znrf1*^Δmye^ myeloid cells compared with controls (Fig. 5b). Additionally, the populations of activated T cells (CD4^+^CD25^+^) and CD4^+^ T_CM_ cells were significantly elevated in *Znrf1*^Δmye^ cultures following MOG_35-55_ stimulation (Fig. 5c and d). We also measured cytokine levels in the co-culture supernatants. IFNγ levels were significantly increased in *Znrf1*^Δmye^ co-cultures after MOG_35-55_ stimulation, whereas IL-17A levels were only slightly elevated (Fig. 5e). By contrast, the levels of pro-inflammatory cytokines, including granulocyte–macrophage colony-stimulating factor (GM-CSF), IL-1β, IL-6, and tumor necrosis factor-α (TNFα), were comparable between *Znrf1*^F/F^ and *Znrf1*^Δmye^ cultures following MOG_35-55_ stimulation (Fig. 5f). Together, these findings demonstrate that ZNRF1 suppresses myeloid cells-mediated antigen-specific T cell proliferation and activation.

### ZNRF1 deficiency upregulates MHC-II on macrophages after EAE induction

**Fig. 6:**
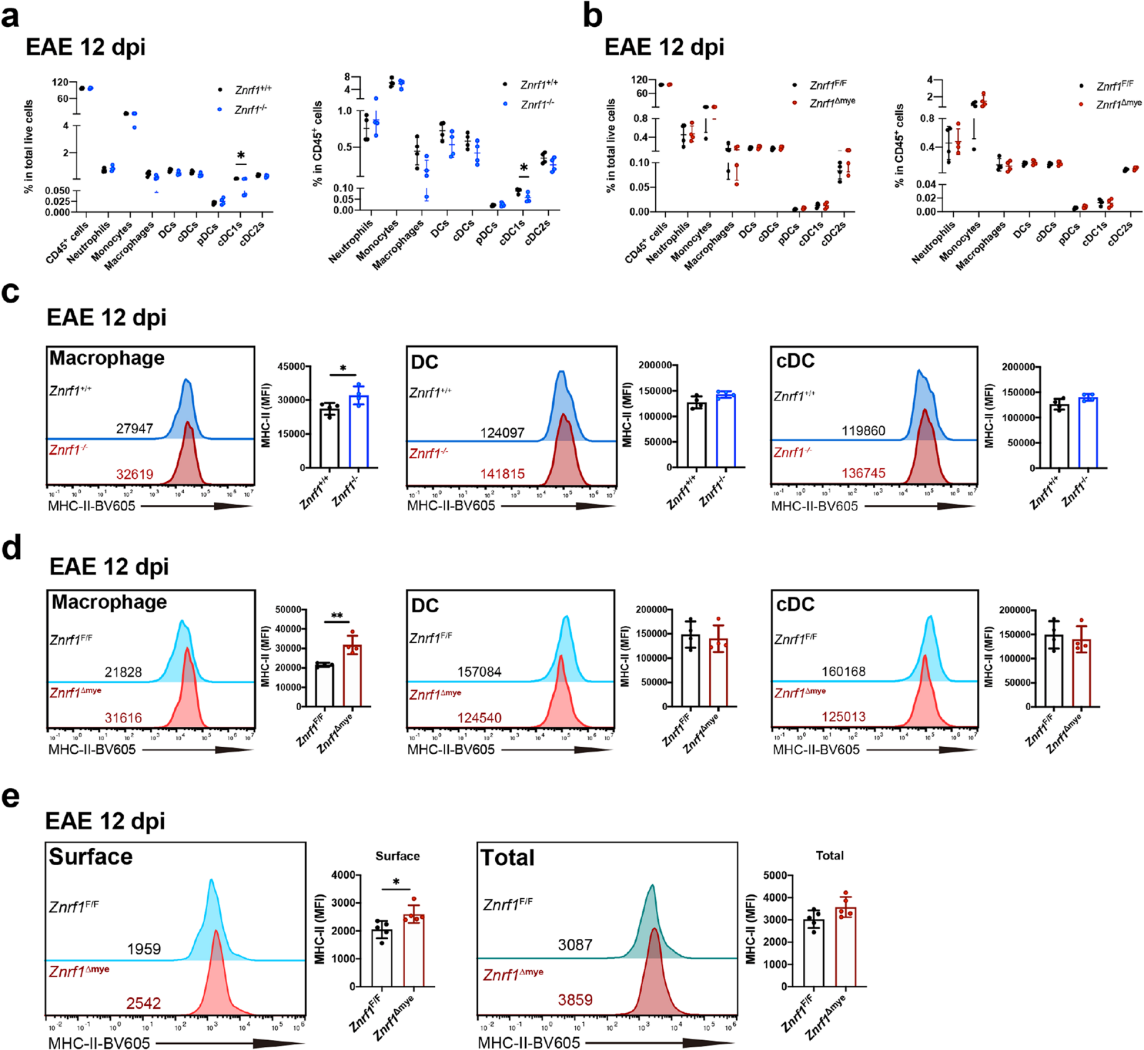
ZNRF1 deficiency in myeloid cells alters macrophage surface MHC-II expression following EAE induction. **a**, **b** Dot plots showing the percentages of CD45^+^cells, neutrophils, monocytes, macrophages, DCs, cDCs, pDCs, cDC1s, and cDC2s within total live cells (left) and within CD45^+^ cells (right) in the spleens from *Znrf1*^+/+^ (N = 4) versus *Znrf1*^−/−^ (*N* = 4) mice (a) or *Znrf1*^F/F^ (*N* = 4) versus *Znrf1*^Δmye^ (*N* = 4) mice (**b**) at day 12 post-EAE induction. **c**, **d** Flow cytometric analysis of surface MHC-II expression on macrophages, DCs, and cDCs in the spleens of *Znrf1*^+/+^ (*N* = 4) versus *Znrf1*^−/−^ (*N* = 4) mice (**c**) or *Znrf1*^F/F^ (*N* = 4) versus *Znrf1*^Δmye^ (*N* = 4) mice (**d**) at day 12 post-EAE induction. **e** Flow cytometric analysis of surface and total MHC-II expression on macrophages in the spleens of *Znrf1*^F/F^ (*N* = 5) versus *Znrf1*^Δmye^ (*N* = 5) mice at day 12 post-EAE induction. The right panels show the quantified mean fluorescence intensity (MFI) of MHC-II. Data are presented as mean ± SD. *P < 0.05, **P < 0.01, determined by the unpaired Student’s *t*-test

Our findings suggest that ZNRF1 deficiency in myeloid cells leads to more severe EAE progression, likely through enhanced antigen-specific T cell proliferation, activation, and differentiation. APCs are known to regulate T cell activation and functions through various surface molecules, including MHC-I, MHC-II, CD40, PD-L1, FasL, and CD80/CD86 [9, 48, 84]. To determine whether ZNRF1 deficiency affects the expression of these regulatory molecules, we performed flow cytometric analysis on spleen-derived myeloid subsets at day 12 post-EAE. We defined and quantified distinct myeloid APC cell subsets, including neutrophils (CD45^+^CD11c^−^B220^−^F4/80^−^ CD11b^+^Ly6G^+^Ly6C^−^), monocytes (CD45^+^CD11c^−^B220^−^F4/80^−^CD11b^+^Ly6G^−^Ly6C^+^), macrophages (CD45^+^Ly6G^−^Ly6C^−^CD11c^−^B220^−^CD11b^+^F4/80^+^), dendritic cells (DCs; CD45^+^Ly6G^−^Ly6C^−^CD11b^−^F4/80^−^CD11c^+^), conventional DCs (cDC; CD45^+^Ly6G^−^Ly6C^−^CD11b^−^F4/80^−^CD11c^+^B220^−^), plasmacytoid DCs (pDC; CD45^+^Ly6G^−^Ly6C^−^CD11b^−^F4/80^−^CD11c^+^B220^+^), cDC1(CD45^+^Ly6G^−^Ly6C^−^CD11b^−^F4/80^−^CD11c^+^B220^−^CD172a^−^XCR1^+^), and cDC2 (CD45^+^Ly6G^−^Ly6C^−^CD11b^−^F4/80^−^CD11c^+^B220^−^CD172a^+^XCR1^−^) (Fig. S7a). We found no significant differences in the percentage of these leukocytes and myeloid APC cell subsets between *Znrf1*^+/+^ vs. *Znrf1*^−/−^ mice (Fig. 6a) or *Znrf1*^F/F^ vs. *Znrf1*^Δmye^ mice (Fig. 6b) at day 12 post-EAE.

Given that MHC-II is crucial for CD4^+^ T cell activation, we measured its surface expression on these myeloid cells. Compared to *Znrf1*^+/+^ mice, *Znrf1*^−/−^ macrophages displayed increased MHC-II surface expression, whereas other myeloid APC subtypes did not, at day 12 after EAE induction (Fig. 6c and S8a). Similarly, *Znrf1*^Δmye^ macrophages, but not other myeloid APC subtypes, showed upregulated MHC-II surface levels compared to *Znrf1*^F/F^ controls at both day 9 and day 12 (Fig. 6d, S7c, and S9a). By contrast, MHC-I levels, which are important for CD8^+^ T cell activation, remained unchanged across all myeloid APC subtypes in both *Znrf1*^+/+^ vs. *Znrf1*^−/−^ (Fig. S8e) and *Znrf1*^F/F^ vs. *Znrf1*^Δmye^ mice (Fig. S9e). Furthermore, surface levels of co-stimulatory molecules (CD40 and CD80/86) and inhibitory molecules (PD-L1 and FasL) were comparable between *Znrf1*^+/+^ vs. *Znrf1*^−/−^ (Fig. S8b-S8d and S10a-S10b) and *Znrf1*^F/F^ vs. *Znrf1*^Δmye^ mice at day 12 post-EAE induction (Fig. S9b-S9d and S10c-S10d). To determine whether ZNRF1 regulates MHC-II protein expression or its surface localization, we assessed both surface and total MHC-II expression on macrophages from *Znrf1*^F/F^ and *Znrf1*^Δmye^ mice at day 12 post-EAE induction. Surface expression of MHC-II, but not MHC-I, FasL, or PD-L1, was significantly increased in *Znrf1*^Δmye^ macrophages compared with *Znrf1*^F/F^ controls (Fig. 6e and Fig. S11). However, total MHC-II levels in macrophages were comparable between *Znrf1*^F/F^ mice and *Znrf1*^Δmye^ groups (Fig. 6e). These findings indicate that ZNRF1 selectively regulates MHC-II surface expression on macrophages during EAE.

In summary, our work reveals that ZNRF1, an E3 ubiquitin ligase, plays a critical role in suppressing the pathogenesis of EAE. Specifically, ZNRF1 deficiency in myeloid cells, but not microglia, leads to exacerbated disease, driven by enhanced antigen-specific T cell activation, proliferation, and Th1/Th17 polarization. Elevated MHC-II surface expression on ZNRF1-deficient macrophages likely underlies this enhanced T cell priming. These findings highlight ZNRF1 as a novel immune checkpoint regulator with potential relevance to MS pathogenesis.

## Discussion

Multiple sclerosis (MS) is a common chronic inflammatory disease of the CNS, characterized demyelination that forms plaques in the brain and spinal cord, accompanied by infiltration of immune cells [83, 88]. The entry of peripheral immune cells, including macrophages, DCs, T cells, and B cells, into the CNS through a disrupted blood–brain barrier (BBB), along with the activation of resident microglia and astrocytes, collectively contributes to inflammation, demyelination, and neuronal damage [37, 65, 83, 88]. However, the precise mechanisms driving neuroinflammation in MS remain incompletely understood. In this study, we demonstrate that ZNRF1 plays a protective role in EAE progression, specifically in peripheral macrophages rather than microglia. ZNRF1 deficiency in myeloid cells led to increased immune cell infiltration into the CNS and more severe EAE clinical symptoms upon MOG_35-55_ immunization. Furthermore, the absence of ZNRF1 in myeloid-derived macrophages enhanced the activation and differentiation of T cells in peripheral lymphoid organs. These data collectively indicate that ZNRF1 regulates MHC-II surface expression in myeloid cells to prevent excessive T cell-mediated neuroinflammation during EAE.

Accumulated evidences indicate that CD4^+^ T cells play a critical role in MS pathogenesis and are closely associated with disease severity [45, 73]. Th1 and Th17 subsets are particularly important in driving MS progression in humans as well as in the murine EAE model, which mimics aspects of MS [45]. Our data suggest that ZNRF1 does not directly involve in CD4^+^ T cell regulations to affect EAE progression. T cell maturation in the thymus and peripheral lymphoid organs appeared normal, and no defects in T cell activation or polarization were observed in *Znrf1*^−/−^ mice. Additionally, the similar disease severity observed in both *Znrf1*^−/−^ mice and *Znrf1*^Δmye^ mice further suggests that ZNRF1’s immunoregulatory role in the EAE model is mediated through myeloid cells, which in turn regulate T cell function.

While T cells are central to MS pathology, other immune cell types, including monocyte-derived macrophages and microglia, also play essential roles in disease progression [68]. Microglia, as CNS-resident macrophages, are key players in CNS development, immune surveillance, and repair [5, 22, 54]. They exert both pro-inflammation and neuroprotective effects. For example, microglia phagocytose myelin debris and secrete neuroprotective and anti-inflammatory factors that promote remyelination during EAE. However, they also contribute to neuroinflammation by releasing proinflammatory cytokines and chemokines, thereby exacerbating demyelination and lesion formation [32]. Monocyte-derived macrophages exert similar dual roles in EAE. They infiltrate the CNS, contribute to axonal degeneration, act as antigen-presenting cells, and drive pathogenic Th1 and Th17 responses [11, 67, 77, 91]**.** In MS lesions, upregulated expression of HLA and CD86 on macrophages has been observed [93]. Moreover, in the EAE model, the activation of both macrophages and microglia correlates with disease progression [36]. These data suggest that both microglia and peripheral macrophages contribute to MS and EAE progression. However, recent studies suggest that the relative contributions of microglia and monocyte-derived macrophages to MS pathogenesis are context-dependent, varying by disease phase and CNS compartment. In the early stages of EAE, macrophages and DCs exhibit stronger antigen-presenting capabilities than microglia. In contrast, in the later phases of EAE, microglia become key APCs stimulating T cells [68]. Microglia are particularly proinflammatory in the early phases of MS but play protective roles in later stages, promoting repair through debris clearance, neuroprotective factors and anti-inflammatory cytokines secretion, and antioxidant production [9, 46, 79]. Supporting this dual role, PLX5622-mediated microglia depletion delays EAE onset but does not affect the chronic disease phase [60]. Conversely, depletion of macrophages reduces CNS damage and disease severity in rat EAE models [10, 34]. Molecular pathways governing macrophages versus microglial function in EAE are being elucidated. For example, Gasdermin D (GSDMD), which triggers pyroptosis, and Signal Transducer and Activator of Transcription 3 (STAT3), a transcription factor in the Janus kinase (JAK)/STAT family, contribute to EAE through peripheral myeloid cells, but not microglia [14, 49]. Cbl-b, a Cbl family E3 ubiquitin ligase, also acts in monocyte-derived macrophages, but not microglia, to inhibit IL-6 production and Th17 differentiation, thus limiting EAE progression [89, 91].

Our study adds to this growing body of evidence by showing that ZNRF1 in peripheral macrophages, but not microglia, regulates EAE progression. Consistently, ZNRF1 deficiency in myeloid cells, but not microglia, led to an increase in pathogenic Th1 and Th17 responses. In contrast, comparable infiltration of Th1 and Th17 cells was observed between wild-type and *Znrf1*^Δglia^ mice, suggesting that the lack of phenotype in *Znrf1*^Δglia^ mice is unlikely due to the absence of other immune APCs required for T cell priming. Furthermore, surface expression of MHC-II was significantly upregulated on peripheral macrophages in *Znrf1*^−/−^ mice, while co-stimulatory molecules such as CD40, CD80, or CD86 remained unchanged. Additionally, myeloid cells isolated from immunized *Znrf1*^Δmye^ mice enhanced the activation and differentiation of CD4^+^ T cells from 2D2 transgenic mice ex vivo. Since MHC-II is essential for antigen presentation to CD4^+^ T cells [71, 81], and MHC-II alleles are strongly associated with MS susceptibility [9, 16, 17], our findings suggest that ZNRF1 is a key regulator of MHC-II surface expression, and, by extension, of T cell activation and neuroinflammation.

The surface expression of MHC-II on myeloid cells is known to increase during inflammation [9, 16, 17]. Our previous studies have shown that ZNRF1 modulates inflammatory responses mediated by TLR4 and TLR3 through distinct mechanisms [47, 50]. ZNRF1 regulates TLR4-driven immune responses by targeting caveolin-1, a major component of caveolae, for proteasomal degradation. On the other hand, ZNRF1 directly ubiquitinates TLR3 to regulate its lysosomal trafficking and termination of downstream signaling. In addition, ZNRF1 activity can also be modulated by receptor-mediated tyrosine kinases, thereby influencing downstream responses [50, 74]. Our data suggest that ZNRF1 suppresses EAE pathogenesis by maintaining homeostasis levels of cell surface MHC-II on macrophages. MHC-II is trafficked to the cell surface via endosomal-lysosomal compartments following synthesis [39, 40, 71]. E3 ubiquitin ligases, such as MARCHI, have been shown to restrict MHC-II level by ubiquitinating its β chain and promoting its degradation [56, 71]. As ZNRF1 is also an E3 ubiquitin ligase, it remains to be determined whether it regulates MHC-II trafficking to the cell surface through ubiquitination. Our findings highlight ZNRF1 as a key regulator of EAE development through its control of MHC-II surface expression on myeloid cells. Further investigations are warranted to elucidate the underlying mechanisms by which inflammatory signals activate ZNRF1 and how ZNRF1, in turn, modulates MHC-II surface expression during EAE.

**Fig. 7:**
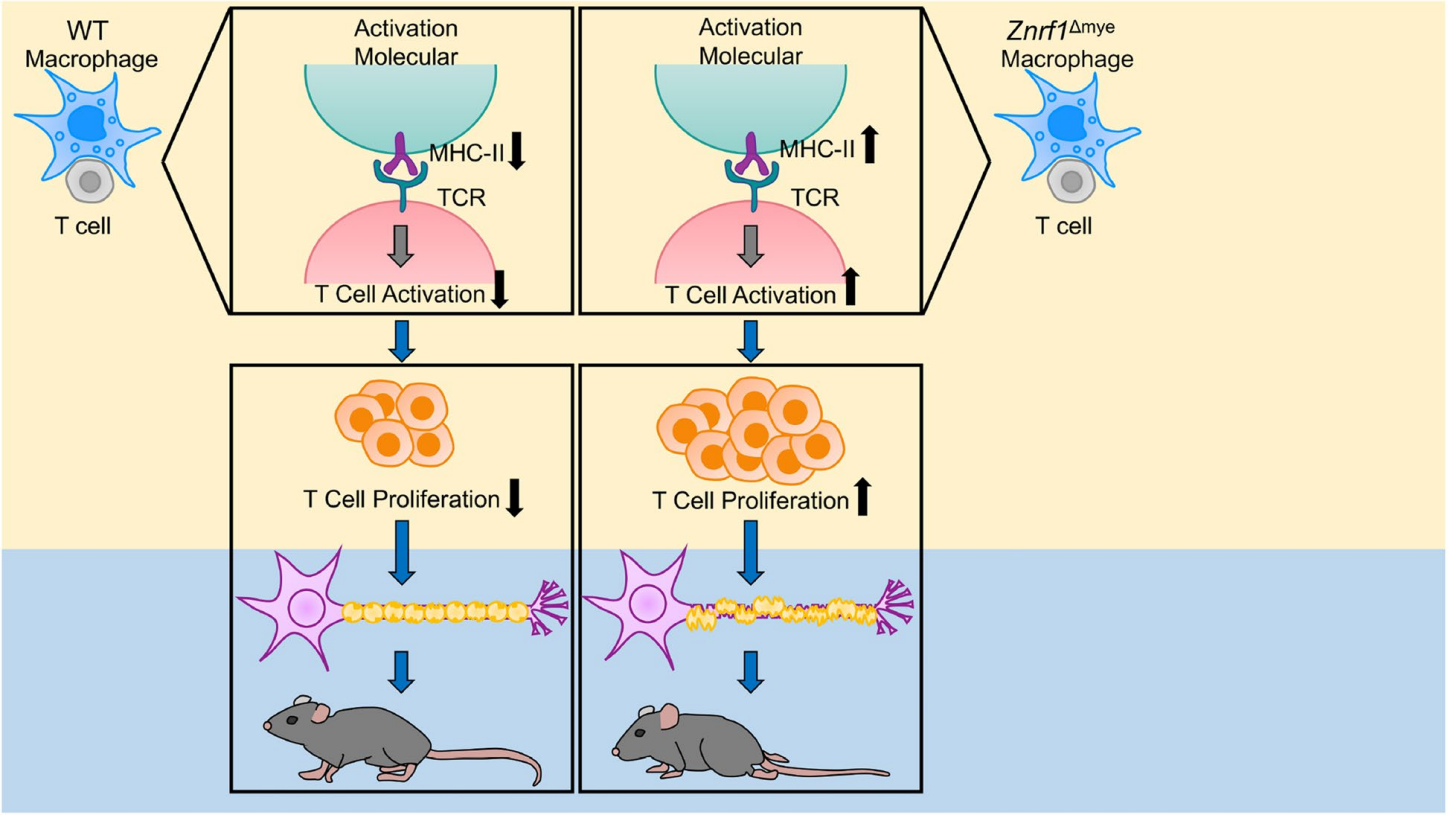
Graphical model summarizing the protective role of ZNRF1 in EAE induction. Proposed schematic illustrating how ZNRF1 in myeloid cells regulates the surface expression of the key regulatory molecule, MHC-II, thereby controlling antigen-specific T cell proliferation and activation. This model highlights the suppressive role of ZNRF1 in myeloid cells during neuroinflammation

In summary, our findings demonstrate that ZNFR1 in peripheral macrophages, but not microglia, plays a crucial immunosuppressive role in autoimmune neuroinflammation and EAE pathogenesis. ZNRF1 maintains the homeostasis of surface MHC-II expression on macrophages, thereby restraining CD4^+^ T cell activation and differentiation. This process attenuates T cell-driven neuroinflammation and demyelination in the CNS during EAE (Fig. 7). These insights identify ZNRF1 as a potential biomarker and therapeutic target for MS, with implications for further modulation of myeloid cell function in neuroinflammatory diseases.

## Supplementary Information

Supplementary Material 1

## Data Availability

No datasets were generated or analysed during the current study.

## Abbreviations

AKT: Protein kinase B
APCs: Antigen-presenting cells
BBB: Blood-brain barrier
BFA: Brefeldin A
BMDMs: Bone marrow-derived macrophages
DCs: Dendritic cells
CFA: Freund’s complete adjuvant
CFSE: Carboxyfluorescein succinimidyl ester
CNS: Central nervous system
DAPI: 4′,6-Diamidino-2-phenylindole
cDCs: Conventional DCs
DLNs: Draining lymph nodes
DPBS: Dulbecco’s phosphate buffer saline
EAE: Experimental autoimmune encephalomyelitis
ECL: Enhanced chemiluminescence
EDTA: Ethylenediaminetetraacetic acid
EGFR: Epidermal growth factor receptor
FasL: Fas ligand
FBS: Fetal bovine serum
PFA: Paraformaldehyde
GSDMD: Gasdermin D
H&E: Hematoxylin and eosin
IHC: Immunohistochemistry
i.p.: Intraperitoneal
JAK: Janus kinase
LFB: Luxol fast blue
Lyve-1: Lymphatic endothelial hyaluronan receptor 1
MFI: Mean fluorescence intensity
MHC-I: Major histocompatibility complex class I
MHC-II: Major histocompatibility complex class II
MOG: Myelin oligodendrocyte glycoprotein
MS: Multiple sclerosis
NaCl: Sodium chloride
NEAA: Nonessential amino acids
PBS: Phosphate buffer saline
pDCs: Plasmacytoid DCs
PD-1: Programmed cell death protein-1
PD-L1: Programmed cell death ligand-1
PNS: Peripheral nervous system
PVDF: Polyvinylidene fluoride
RBC: Red blood cell
RIPA: Radioimmunoprecipitation assay buffer
RRMS: Relapsing-remitting multiple sclerosis
RPMI: Roswell Park Memorial Institute
RIPA: Radioimmunoprecipitation assay
SDS: Sodium dodecyl sulfate
STAT3: Signal transducer and activator of transcription 3
SPMS: Secondary progressive multiple sclerosis
T_CM_: Central memory T
T_EM_: Effector memory T
Th: T helper
Th1: Interferon (IFN)-γ-producing CD4^+^ T
Th17: Interleukin-17 (IL-17)-producing CD4^+^ T
TLR3: Toll-like receptor 3
TLR4: Toll-like receptor 4
Tris: Tris (hydroxymethyl) aminomethane
WGA: Wheat germ agglutinin
WT: Wild-type
ZNRF1: Zinc and RING finger 1

## References

[1] Akaishi T, Takahashi T, Nakashima I. Peripheral blood monocyte count at onset may affect the prognosis in multiple sclerosis. J Neuroimmunol. 2018;319:37–40. 29685288 10.1016/j.jneuroim.2018.03.016

[2] Alfredsson L, Olsson T. Lifestyle and environmental factors in multiple sclerosis. Cold Spring Harb Perspect Med. 2019. 10.1101/cshperspect.a028944. 29735578 10.1101/cshperspect.a028944 PMC6444694

[3] Araki T, Milbrandt J. ZNRF proteins constitute a family of presynaptic E3 ubiquitin ligases. J Neurosci. 2003;23(28):9385–94. 14561866 10.1523/JNEUROSCI.23-28-09385.2003 PMC6740566

[4] Araki T, Nagarajan R, Milbrandt J. Identification of genes induced in peripheral nerve after injury. Expression profiling and novel gene discovery. J Biol Chem. 2001;276(36):34131–41. 11427537 10.1074/jbc.M104271200

[5] Attfield KE, Jensen LT, Kaufmann M, Friese MA, Fugger L. The immunology of multiple sclerosis. Nat Rev Immunol. 2022;22(12):734–50. 35508809 10.1038/s41577-022-00718-z

[6] Baaten BJ, Cooper AM, Swain SL, Bradley LM. Location, location, location: the impact of migratory heterogeneity on T cell function. Front Immunol. 2013;4:311. 24115949 10.3389/fimmu.2013.00311 PMC3792444

[7] Babbe H, Roers A, Waisman A, Lassmann H, Goebels N, Hohlfeld R, et al. Clonal expansions of CD8+ T cells dominate the T cell infiltrate in active multiple sclerosis lesions as shown by micromanipulation and single cell polymerase chain reaction. J Exp Med. 2000;192(3):393–404. 10934227 10.1084/jem.192.3.393 PMC2193223

[8] Bankhead P, Loughrey MB, Fernandez JA, Dombrowski Y, McArt DG, Dunne PD, et al. QuPath: Open source software for digital pathology image analysis. Sci Rep. 2017;7(1):16878. 29203879 10.1038/s41598-017-17204-5 PMC5715110

[9] Baranzini SE, Oksenberg JR. The genetics of multiple sclerosis: from 0 to 200 in 50 years. Trends Genet. 2017;33(12):960–70. 28987266 10.1016/j.tig.2017.09.004 PMC5701819

[10] Brosnan CF, Bornstein M, Bloom BR. The effects of macrophage depletion on the clinical and pathologic expression of experimental allergic encephalomyelitis. J Immunol. 1981;126(2):614–20. 6256443

[11] Brück W, Porada P, Poser S, Rieckmann P, Hanefeld F, Kretzschmarch HA, et al. Monocyte/macrophage differentiation in early multiple sclerosis lesions. Ann Neurol. 1995;38(5):788–96. 7486871 10.1002/ana.410380514

[12] Carswell R. Pathological anatomy: illustrations of the elementary forms of disease. Orme, Brown, Green and Longman: Longman; 1838.

[13] Charcot J.M. Lectures on the diseases of the nervous system, HC Lea, 1879.

[14] Chen CC, Peng SJ, Wu PY, Chien HJ, Lee CY, Chung MH, et al. Heterogeneity and neurovascular integration of intraportally transplanted islets revealed by 3-d mouse liver histology. Am J Physiol Endocrinol Metab. 2021;320(6):E1007–19. 33900850 10.1152/ajpendo.00605.2020

[15] Clausen BE, Burkhardt C, Reith W, Renkawitz R, Forster I. Conditional gene targeting in macrophages and granulocytes using LysMcre mice. Transgenic Res. 1999;8(4):265–77. 10621974 10.1023/a:1008942828960

[16] Compston A, Coles A. Multiple sclerosis. Lancet. 2002;359(9313):1221–31. 11955556 10.1016/S0140-6736(02)08220-X

[17] Consortium I.M.S.G. Risk alleles for multiple sclerosis identified by a genomewide study. N Engl J Med. 2007;357(9):851–62. 17660530 10.1056/NEJMoa073493

[18] Constantinescu CS, Farooqi N, O’Brien K, Gran B. Experimental autoimmune encephalomyelitis (EAE) as a model for multiple sclerosis (MS). Br J Pharmacol. 2011;164(4):1079–106. 21371012 10.1111/j.1476-5381.2011.01302.x PMC3229753

[19] Cruciani C, Puthenparampil M, Tomas-Ojer P, Jelcic I, Docampo MJ, Planas R, et al. T-cell specificity influences disease heterogeneity in multiple sclerosis. Neurol Neuroimmunol Neuroinflamm. 2021. 10.1212/NXI.0000000000001075. 34535569 10.1212/NXI.0000000000001075 PMC8453544

[20] Dowling P, Husar W, Menonna J, Donnenfeld H, Cook S, Sidhu M. Cell death and birth in multiple sclerosis brain. J Neurol Sci. 1997;149(1):1–11. 9168159 10.1016/s0022-510x(97)05213-1

[21] Duffy SS, Lees JG, Moalem-Taylor G. The contribution of immune and glial cell types in experimental autoimmune encephalomyelitis and multiple sclerosis. Mult Scler Int. 2014;2014:285245. 25374694 10.1155/2014/285245 PMC4211315

[22] Espinosa-Parrilla J, Pugliese M, Mahy N and Rodríguez MJ. Neuroprotection: A New Therapeutic Approach of Relapsing Remitting Multiple Sclerosis. Trending Topics in Multiple Sclerosis, 2016.

[23] Filippi M, Bar-Or A, Piehl F, Preziosa P, Solari A, Vukusic S, et al. Multiple sclerosis. Nat Rev Dis Primers. 2018;4(1):43. 30410033 10.1038/s41572-018-0041-4

[24] Fissolo N, Haag S, de Graaf KL, Drews O, Stevanovic S, Rammensee HG, et al. Naturally presented peptides on major histocompatibility complex I and II molecules eluted from central nervous system of multiple sclerosis patients. Mol Cell Proteomics. 2009;8(9):2090–101. 19531498 10.1074/mcp.M900001-MCP200 PMC2742442

[25] Ganguly D, Haak S, Sisirak V, Reizis B. The role of dendritic cells in autoimmunity. Nat Rev Immunol. 2013;13(8):566–77. 23827956 10.1038/nri3477 PMC4160805

[26] Gilmore CP, Donaldson I, Bo L, Owens T, Lowe J, Evangelou N. Regional variations in the extent and pattern of grey matter demyelination in multiple sclerosis: a comparison between the cerebral cortex, cerebellar cortex, deep grey matter nuclei and the spinal cord. J Neurol Neurosurg Psychiatry. 2009;80(2):182–7. 18829630 10.1136/jnnp.2008.148767

[27] Ginhoux F, Greter M, Leboeuf M, Nandi S, See P, Gokhan S, et al. Fate mapping analysis reveals that adult microglia derive from primitive macrophages. Science. 2010;330(6005):841–5. 20966214 10.1126/science.1194637 PMC3719181

[28] Gomes AC, Morris M, Stawiarz L, Jonsson G, Putheti P, Bronge L, et al. Decreased levels of CD95 and caspase-8 mRNA in multiple sclerosis patients with gadolinium-enhancing lesions on MRI. Neurosci Lett. 2003;352(2):101–4. 14625033 10.1016/j.neulet.2003.08.030

[29] Gou Q, Dong C, Xu H, Khan B, Jin J, Liu Q, et al. PD-L1 degradation pathway and immunotherapy for cancer. Cell Death Dis. 2020;11(11):955. 33159034 10.1038/s41419-020-03140-2 PMC7648632

[30] Goverman J. Autoimmune T cell responses in the central nervous system. Nat Rev Immunol. 2009;9(6):393–407. 19444307 10.1038/nri2550 PMC2813731

[31] Greter M, Heppner FL, Lemos MP, Odermatt BM, Goebels N, Laufer T, et al. Dendritic cells permit immune invasion of the CNS in an animal model of multiple sclerosis. Nat Med. 2005;11(3):328–34. 15735653 10.1038/nm1197

[32] Heppner FL, Greter M, Marino D, Falsig J, Raivich G, Hövelmeyer N, et al. Experimental autoimmune encephalomyelitis repressed by microglial paralysis. Nat Med. 2005;11(2):146–52. 15665833 10.1038/nm1177

[33] Huang Y, Xu Z, Xiong S, Sun F, Qin G, Hu G, et al. Repopulated microglia are solely derived from the proliferation of residual microglia after acute depletion. Nat Neurosci. 2018;21(4):530–40. 29472620 10.1038/s41593-018-0090-8

[34] Huitinga I, Van Rooijen N, De Groot C, Uitdehaag B, Dijkstra C. Suppression of experimental allergic encephalomyelitis in Lewis rats after elimination of macrophages. J Exp Med. 1990;172(4):1025–33. 2145387 10.1084/jem.172.4.1025 PMC2188611

[35] Jacobs BM, Noyce AJ, Bestwick J, Belete D, Giovannoni G, Dobson R. Gene-environment interactions in multiple sclerosis: a UK biobank study. Neurol Neuroimmunol Neuroinflamm. 2021. 10.1212/NXI.0000000000001007. 34049995 10.1212/NXI.0000000000001007 PMC8192056

[36] Jiang Z, Jiang JX, Zhang G-X. Macrophages: a double-edged sword in experimental autoimmune encephalomyelitis. Immunol Lett. 2014;160(1):17–22. 24698730 10.1016/j.imlet.2014.03.006 PMC6186449

[37] Jung S, Schwartz M. Non-identical twins - microglia and monocyte-derived macrophages in acute injury and autoimmune inflammation. Front Immunol. 2012;3:89. 22566968 10.3389/fimmu.2012.00089 PMC3345364

[38] Karni A, Abraham M, Monsonego A, Cai G, Freeman GJ, Hafler D, et al. Innate immunity in multiple sclerosis: myeloid dendritic cells in secondary progressive multiple sclerosis are activated and drive a proinflammatory immune response. J Immunol. 2006;177(6):4196–202. 16951385 10.4049/jimmunol.177.6.4196

[39] Kleijmeer MJ, Oorschot VM, Geuze HJ. Human resident langerhans cells display a lysosomal compartment enriched in MHC class II. J Invest Dermatol. 1994;103(4):516–23. 7930676 10.1111/1523-1747.ep12395666

[40] Kleijmeer MJ, Ossevoort MA, van Veen CJ, van Hellemond JJ, Neefjes JJ, Kast WM, et al. MHC class II compartments and the kinetics of antigen presentation in activated mouse spleen dendritic cells. J Immunol. 1995;154(11):5715–24. 7751623

[41] Kouwenhoven M, Teleshova N, Ozenci V, Press R, Link H. Monocytes in multiple sclerosis: phenotype and cytokine profile. J Neuroimmunol. 2001;112(1–2):197–205. 11108949 10.1016/s0165-5728(00)00396-9

[42] Krogsgaard M, Wucherpfennig KW, Cannella B, Hansen BE, Svejgaard A, Pyrdol J, et al. Visualization of myelin basic protein (MBP) T cell epitopes in multiple sclerosis lesions using a monoclonal antibody specific for the human histocompatibility leukocyte antigen (HLA)-DR2-MBP 85–99 complex. J Exp Med. 2000;191(8):1395–412. 10770805 10.1084/jem.191.8.1395 PMC2193136

[43] Kroner A, Mehling M, Hemmer B, Rieckmann P, Toyka KV, Mäurer M, et al. A PD-1 polymorphism is associated with disease progression in multiple sclerosis. Ann Neurol. 2005;58(1):50–7. 15912506 10.1002/ana.20514

[44] Kryczek I, Zhao E, Liu Y, Wang Y, Vatan L, Szeliga W, et al. Human TH17 cells are long-lived effector memory cells. Sci Transl Med. 2011;3(104):104ra100. 21998407 10.1126/scitranslmed.3002949 PMC3345568

[45] Kunkl M, Frascolla S, Amormino C, Volpe E, Tuosto L. T helper cells: the modulators of inflammation in multiple sclerosis. Cells. 2020. 10.3390/cells9020482. 32093011 10.3390/cells9020482 PMC7072830

[46] Kwilasz AJ, Grace PM, Serbedzija P, Maier SF, Watkins LR. The therapeutic potential of interleukin-10 in neuroimmune diseases. Neuropharmacology. 2015;96(Pt A):55–69. 25446571 10.1016/j.neuropharm.2014.10.020 PMC5144739

[47] Lee CY, Lai TY, Tsai MK, Chang YC, Ho YH, Yu IS, et al. The ubiquitin ligase ZNRF1 promotes caveolin-1 ubiquitination and degradation to modulate inflammation. Nat Commun. 2017;8:15502. 28593998 10.1038/ncomms15502 PMC5472178

[48] Li H, Zheng C, Han J, Zhu J, Liu S, Jin T. PD-1/PD-L1 axis as a potential therapeutic target for multiple sclerosis: a T cell perspective. Front Cell Neurosci. 2021;15:716747. 34381337 10.3389/fncel.2021.716747 PMC8350166

[49] Li S, Wu Y, Yang D, Wu C, Ma C, Liu X, et al. Gasdermin d in peripheral myeloid cells drives neuroinflammation in experimental autoimmune encephalomyelitis. J Exp Med. 2019;216(11):2562–81. 31467036 10.1084/jem.20190377 PMC6829591

[50] Lin YS, Chang YC, Chao TL, Tsai YM, Jhuang SJ, Ho YH, et al. The Src-ZNRF1 axis controls TLR3 trafficking and interferon responses to limit lung barrier damage. J Exp Med. 2023. 10.1084/jem.20220727. 37158982 10.1084/jem.20220727 PMC10174191

[51] Liu YA, Chen Y, Chiang AS, Peng SJ, Pasricha PJ, Tang SC. Optical clearing improves the imaging depth and signal-to-noise ratio for digital analysis and three-dimensional projection of the human enteric nervous system. Neurogastroenterol Motil. 2011;23(10):e446-457. 21895876 10.1111/j.1365-2982.2011.01773.x

[52] Lock C, Hermans G, Pedotti R, Brendolan A, Schadt E, Garren H, et al. Gene-microarray analysis of multiple sclerosis lesions yields new targets validated in autoimmune encephalomyelitis. Nat Med. 2002;8(5):500–8. 11984595 10.1038/nm0502-500

[53] Lodygin D, Hermann M, Schweingruber N, Flugel-Koch C, Watanabe T, Schlosser C, et al. Beta-Synuclein-reactive T cells induce autoimmune CNS grey matter degeneration. Nature. 2019;566(7745):503–8. 30787438 10.1038/s41586-019-0964-2

[54] London A, Cohen M, Schwartz M. Microglia and monocyte-derived macrophages: functionally distinct populations that act in concert in CNS plasticity and repair. Front Cell Neurosci. 2013;7:34. 23596391 10.3389/fncel.2013.00034 PMC3625831

[55] Machado-Santos J, Saji E, Tröscher AR, Paunovic M, Liblau R, Gabriely G, et al. The compartmentalized inflammatory response in the multiple sclerosis brain is composed of tissue-resident CD8+ T lymphocytes and B cells. Brain. 2018;141(7):2066–82. 29873694 10.1093/brain/awy151 PMC6022681

[56] Matsuki Y, Ohmura-Hoshino M, Goto E, Aoki M, Mito-Yoshida M, Uematsu M, et al. Novel regulation of MHC class II function in B cells. EMBO J. 2007;26(3):846–54. 17255932 10.1038/sj.emboj.7601556 PMC1794403

[57] McFarlin DE, McFarland HF. Multiple sclerosis (first of two parts). N Engl J Med. 1982;307(19):1183–8. 6750404 10.1056/NEJM198211043071905

[58] McMahon EJ, Bailey SL, Castenada CV, Waldner H, Miller SD. Epitope spreading initiates in the CNS in two mouse models of multiple sclerosis. Nat Med. 2005;11(3):335–9. 15735651 10.1038/nm1202

[59] Merson TD, Binder MD, Kilpatrick TJ. Role of cytokines as mediators and regulators of microglial activity in inflammatory demyelination of the CNS. Neuromolecular Med. 2010;12(2):99–132. 20411441 10.1007/s12017-010-8112-z

[60] Montilla A, Zabala A, Er-Lukowiak M, Rissiek B, Magnus T, Rodriguez-Iglesias N, et al. Microglia and meningeal macrophages depletion delays the onset of experimental autoimmune encephalomyelitis. Cell Death Dis. 2023;14(1):16. 36635255 10.1038/s41419-023-05551-3 PMC9835747

[61] Murphy AC, Lalor SJ, Lynch MA, Mills KH. Infiltration of Th1 and Th17 cells and activation of microglia in the CNS during the course of experimental autoimmune encephalomyelitis. Brain Behav Immun. 2010;24(4):641–51. 20138983 10.1016/j.bbi.2010.01.014

[62] Nutt SL, Chopin M. Transcriptional networks driving dendritic cell differentiation and function. Immunity. 2020;52(6):942–56. 32553180 10.1016/j.immuni.2020.05.005

[63] Olsson T, Barcellos LF, Alfredsson L. Interactions between genetic, lifestyle and environmental risk factors for multiple sclerosis. Nat Rev Neurol. 2017;13(1):25–36. 27934854 10.1038/nrneurol.2016.187

[64] Ortler S, Leder C, Mittelbronn M, Zozulya AL, Knolle PA, Chen L, et al. B7–H1 restricts neuroantigen-specific T cell responses and confines inflammatory CNS damage: implications for the lesion pathogenesis of multiple sclerosis. Eur J Immunol. 2008;38(6):1734–44. 18421793 10.1002/eji.200738071

[65] Passaro AP, Lebos AL, Yao Y, Stice SL. Immune response in neurological pathology: emerging role of central and peripheral immune crosstalk. Front Immunol. 2021;12:676621. 34177918 10.3389/fimmu.2021.676621 PMC8222736

[66] Petrova N, Carassiti D, Altmann DR, Baker D, Schmierer K. Axonal loss in the multiple sclerosis spinal cord revisited. Brain Pathol. 2018;28(3):334–48. 28401686 10.1111/bpa.12516 PMC8028682

[67] Popescu BF, Lucchinetti CF. Pathology of demyelinating diseases. Annu Rev Pathol. 2012;7:185–217. 22313379 10.1146/annurev-pathol-011811-132443

[68] Radandish M, Khalilian P, Esmaeil N. The role of distinct subsets of macrophages in the pathogenesis of MS and the impact of different therapeutic agents on these populations. Front Immunol. 2021;12:667705. 34489926 10.3389/fimmu.2021.667705 PMC8417824

[69] Raphael I, Joern RR, Forsthuber TG. Memory CD4(+) T cells in immunity and autoimmune diseases. Cells. 2020. 10.3390/cells9030531. 32106536 10.3390/cells9030531 PMC7140455

[70] Ravnic DJ, Jiang X, Wolloscheck T, Pratt JP, Huss H, Mentzer SJ, et al. Vessel painting of the microcirculation using fluorescent lipophilic tracers. Microvasc Res. 2005;70(1–2):90–6. 16095629 10.1016/j.mvr.2005.06.002

[71] Roche PA, Furuta K. The ins and outs of MHC class II-mediated antigen processing and presentation. Nat Rev Immunol. 2015;15(4):203–16. 25720354 10.1038/nri3818 PMC6314495

[72] Sahasrabuddhe V, Ghosh HS. Cx3Cr1-Cre induction leads to microglial activation and IFN-1 signaling caused by DNA damage in early postnatal brain. Cell Rep. 2022;38(3):110252. 35045285 10.1016/j.celrep.2021.110252

[73] Schafflick D, Xu CA, Hartlehnert M, Cole M, Schulte-Mecklenbeck A, Lautwein T, et al. Integrated single cell analysis of blood and cerebrospinal fluid leukocytes in multiple sclerosis. Nat Commun. 2020;11(1):247. 31937773 10.1038/s41467-019-14118-w PMC6959356

[74] Shen CH, Chou CC, Lai TY, Hsu JE, Lin YS, Liu HY, et al. ZNRF1 mediates epidermal growth factor receptor ubiquitination to control receptor lysosomal trafficking and degradation. Front Cell Dev Biol. 2021;9:642625. 33996800 10.3389/fcell.2021.642625 PMC8118649

[75] Sie C, Korn T. Dendritic cells in central nervous system autoimmunity. Semin Immunopathol. 2017;39(2):99–111. 27888330 10.1007/s00281-016-0608-7

[76] Singh S, Metz I, Amor S, van der Valk P, Stadelmann C, Bruck W. Microglial nodules in early multiple sclerosis white matter are associated with degenerating axons. Acta Neuropathol. 2013;125(4):595–608. 23354834 10.1007/s00401-013-1082-0 PMC3611040

[77] Singh S, Metz I, Amor S, van der Valk P, Stadelmann C, Brück W. Microglial nodules in early multiple sclerosis white matter are associated with degenerating axons. Acta Neuropathol. 2013;125:595–608. 23354834 10.1007/s00401-013-1082-0 PMC3611040

[78] Takeda K, Clausen BE, Kaisho T, Tsujimura T, Terada N, Forster I, et al. Enhanced Th1 activity and development of chronic enterocolitis in mice devoid of Stat3 in macrophages and neutrophils. Immunity. 1999;10(1):39–49. 10023769 10.1016/s1074-7613(00)80005-9

[79] Tanaka T, Yoshida S. Mechanisms of remyelination: recent insight from experimental models. Biomol Concepts. 2014;5(4):289–98. 25372760 10.1515/bmc-2014-0015

[80] Trapp BD, Peterson J, Ransohoff RM, Rudick R, Mork S, Bo L. Axonal transection in the lesions of multiple sclerosis. N Engl J Med. 1998;338(5):278–85. 9445407 10.1056/NEJM199801293380502

[81] Trombetta ES, Mellman I. Cell biology of antigen processing in vitro and in vivo. Annu Rev Immunol. 2005;23:975–1028. 15771591 10.1146/annurev.immunol.22.012703.104538

[82] Valli A, Sette A, Kappos L, Oseroff C, Sidney J, Miescher G, et al. Binding of myelin basic protein peptides to human histocompatibility leukocyte antigen class II molecules and their recognition by T cells from multiple sclerosis patients. J Clin Invest. 1993;91(2):616–28. 7679413 10.1172/JCI116242 PMC287995

[83] Voet S, Prinz M, van Loo G. Microglia in central nervous system inflammation and multiple sclerosis pathology. Trends Mol Med. 2019;25(2):112–23. 30578090 10.1016/j.molmed.2018.11.005

[84] Volpe E, Sambucci M, Battistini L, Borsellino G. Fas-Fas ligand: checkpoint of T cell functions in multiple sclerosis. Front Immunol. 2016;7:382. 27729910 10.3389/fimmu.2016.00382 PMC5037862

[85] Wakatsuki S, Furuno A, Ohshima M, Araki T. Oxidative stress-dependent phosphorylation activates ZNRF1 to induce neuronal/axonal degeneration. J Cell Biol. 2015;211(4):881–96. 26572622 10.1083/jcb.201506102 PMC4657170

[86] Wakatsuki S, Saitoh F, Araki T. ZNRF1 promotes Wallerian degeneration by degrading AKT to induce GSK3B-dependent CRMP2 phosphorylation. Nat Cell Biol. 2011;13(12):1415–23. 22057101 10.1038/ncb2373

[87] Wang J, Wang J, Wang J, Yang B, Weng Q, He Q. Targeting microglia and macrophages: a potential treatment strategy for multiple sclerosis. Front Pharmacol. 2019;10:286. 30967783 10.3389/fphar.2019.00286 PMC6438858

[88] Woo MS, Engler JB, Friese MA. The neuropathobiology of multiple sclerosis. Nat Rev Neurosci. 2024;25(7):493–513. 38789516 10.1038/s41583-024-00823-z

[89] Yamasaki R, Lu H, Butovsky O, Ohno N, Rietsch AM, Cialic R, et al. Differential roles of microglia and monocytes in the inflamed central nervous system. J Exp Med. 2014;211(8):1533–49. 25002752 10.1084/jem.20132477 PMC4113947

[90] Yona S, Kim KW, Wolf Y, Mildner A, Varol D, Breker M, et al. Fate mapping reveals origins and dynamics of monocytes and tissue macrophages under homeostasis. Immunity. 2013;38(1):79–91. 23273845 10.1016/j.immuni.2012.12.001 PMC3908543

[91] Zeng Q, Tang N, Ma Y, Guo H, Zhao Y, Tang R, et al. Cbl-b restrains priming of pathogenic Th17 cells via the inhibition of IL-6 production by macrophages. iScience. 2022. 10.1016/j.isci.2022.105151. 36185364 10.1016/j.isci.2022.105151 PMC9523381

[92] Zhang J, Bu X, Wang H, Zhu Y, Geng Y, Nihira NT, et al. Cyclin D-CDK4 kinase destabilizes PD-L1 via cullin 3-SPOP to control cancer immune surveillance. Nature. 2018;553(7686):91–5. 29160310 10.1038/nature25015 PMC5754234

[93] Zrzavy T, Hametner S, Wimmer I, Butovsky O, Weiner HL, Lassmann H. Loss of ‘homeostatic’microglia and patterns of their activation in active multiple sclerosis. Brain. 2017;140(7):1900–13. 28541408 10.1093/brain/awx113 PMC6057548

