# Supplementary material for: Myeloid ZNRF1 suppresses autoimmune demyelination and neuroinflammation by regulating MHC-II-mediated T cell activation
Source: J Neuroinflammation. 2025 Oct 22;22:239. doi: 10.1186/s12974-025-03550-z (PMC12542093; doi:10.1186/s12974-025-03550-z)
Supplement: Supplementary file 1 — Supplementary Material 1 [file 12974_2025_3550_MOESM1_ESM.docx]

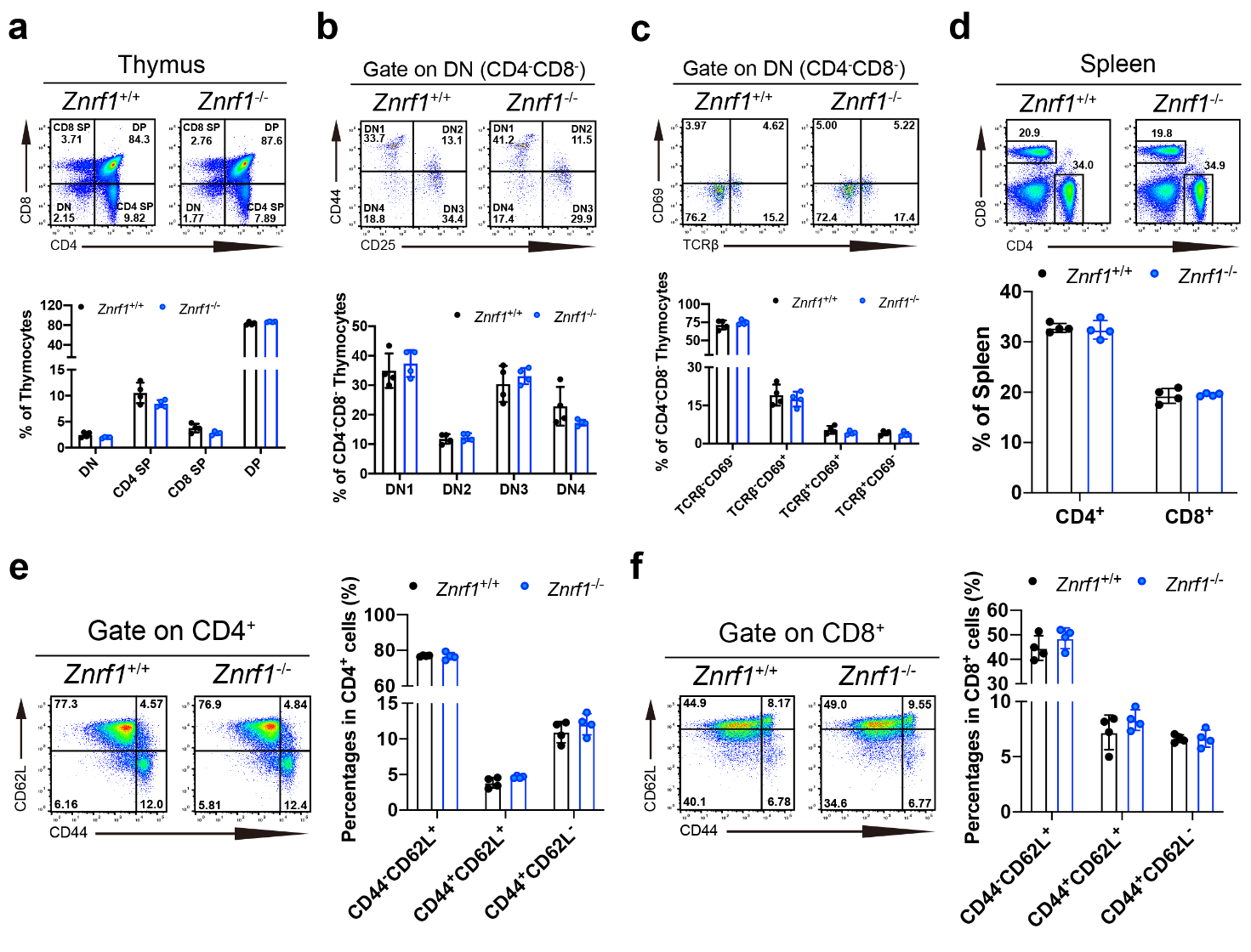


**Supplementary Figure 1. ZNRF1 is dispensable for T-lymphocyte development.**

(a) Flow cytometric analysis of the proportions of double-negative (DN, CD4^-^ CD8^-^), single-positive (CD4 SP, CD4^+^CD8^-^ and CD8 SP, CD4^-^ CD8^+^) and double-positive (DP, CD4^+^ CD8^+^) T cells in the thymus of naïve *Znrf1*^+/+^ (N=4) and *Znrf1*^-/-^ mice (N=4). (b) Flow cytometric analysis of the populations, including DN1 (CD25^-^CD44^+^), DN2 (CD25^+^CD44^+^), DN3 (CD25^+^CD44^-^), and DN4 (CD25^-^CD44^-^), within the DN (CD4^-^CD8^-^) population in the thymus of naïve *Znrf1*^+/+^ (N=4) and *Znrf1*^-/-^ mice (N=4). (c) Flow cytometric analysis of the expression of TCRβ and CD69 within DN T cells in the thymus of naïve *Znrf1*^+/+^ (N=4) and *Znrf1*^-/-^ mice (N=4). (d) Flow cytometric analysis of CD4^+^ and CD8^+^ T cells in the spleens of naïve *Znrf1*^+/+^ (N=4) and *Znrf1*^-/-^ mice (N=4). (e) Flow cytometric analysis of naïve (CD44^-^CD62L^+^), central memory (CD44^+^CD62L^+^), and effector memory (CD44^+^CD62L^-^) CD4^+^ T cells in the spleens of naïve *Znrf1*^+/+^ (N=4) and *Znrf1*^-/-^ mice (N=4). (f) Flow cytometric analysis of naïve (CD44^-^CD62L^+^), central memory (CD44^+^CD62L^+^), and effector memory (CD44^+^CD62L^-^) CD8^+^ T cells in the spleens of naïve *Znrf1*^+/+^ (N=4) and *Znrf1*^-/-^ mice (N=4). Representative flow plots and quantified cell numbers in the spleen and thymus are shown for all panels. Data are presented as mean ± SD. ns, not significant. Statistical significance was determined by the unpaired Student’s *t*-test.


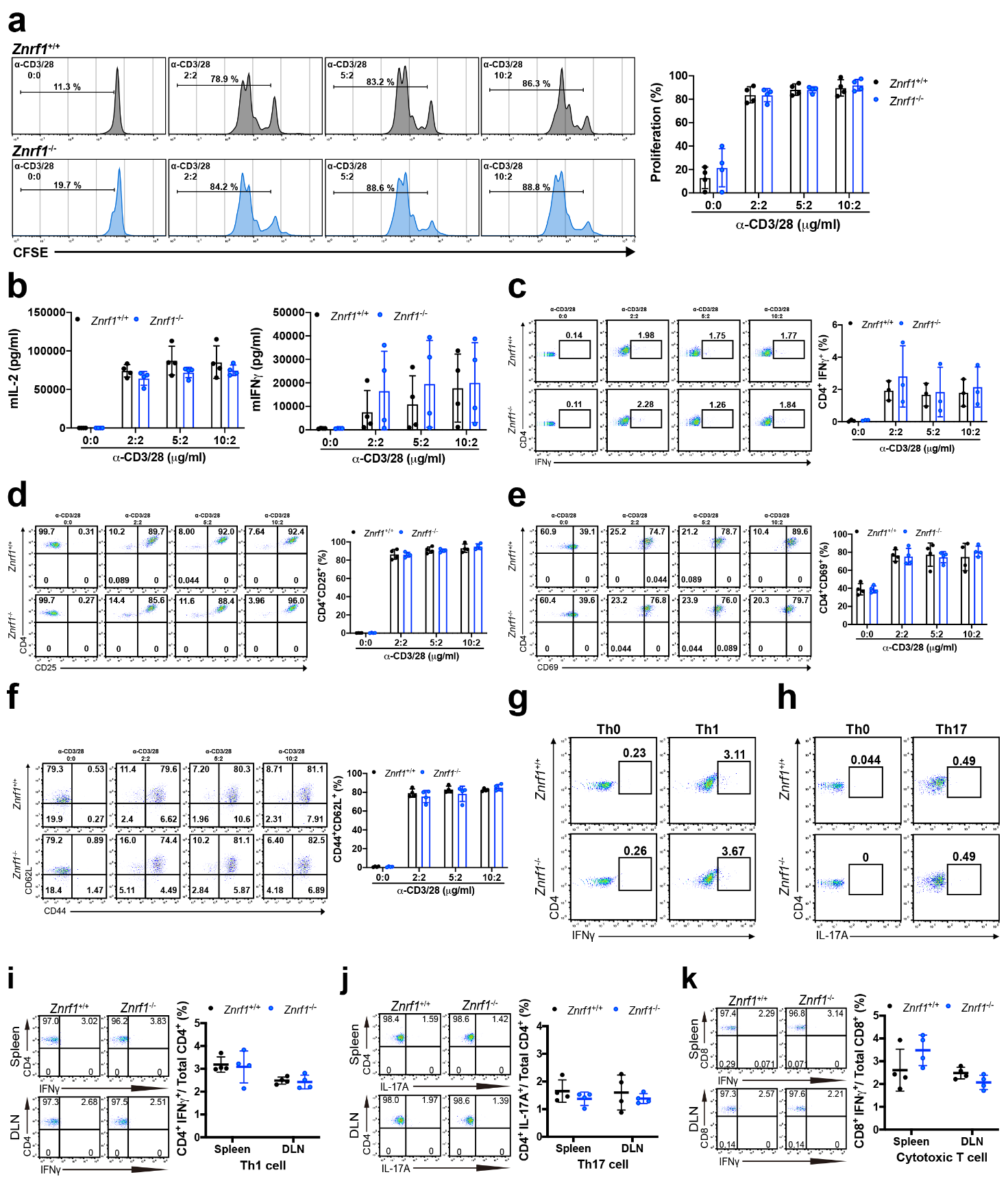


**Supplementary Figure 2. ZNRF1 is dispensable for T cell activation.**

(a-f) CD4^+^ T cells from naive *Znrf1*^+/+^ (N=4) and *Znrf1*^-/-^ mice (N=4) were isolated from the spleens using magnetic selection and stimulated with plate-bound anti-CD3 and soluble anti-CD28 antibodies for 3 days. (a) CD4^+^ T cells were labeled with 5 μM CFSE to assess proliferation. Representative flow plots and quantification of the percentage of proliferating CD4^+^ T cells are shown. (b) CD4^+^ T cells were stimulated with anti-CD3/CD28 antibodies for 3 days, and IL-2 and IFNγ secretion was measured by ELISA. (c-f) Flow cytometric analysis of activated T cells (CD4^+^IFNγ^+^, CD4^+^CD25^+^, CD4^+^CD69^+^, and CD44^+^CD62L^+^) among CD4^+^ T cells in *Znrf1*^+/+^ (N=4) and *Znrf1*^-/-^ mice (N=4), 3 days after anti-CD3/28 antibody stimulation. (g-h) CD4^+^ T cells from naive *Znrf1*^+/+^ and *Znrf1*^-/-^ mice were isolated from spleens using magnetic selection and stimulated under Th1- and Th17-polarizing conditions. (i-k) Flow cytometric analysis of Th1 cells (CD4⁺IFNγ⁺) (i), Th17 cells (CD4⁺IL-17A⁺) (j), and cytotoxic T cells (CD8^+^IFNγ^+^) (k) in spleens and DLNs of naïve *Znrf1*^+/+^ (N=4) and *Znrf1*^-/-^ mice (N=4). Data are presented as mean ± SD. ns, not significant. Statistical significance was determined by the unpaired Student’s *t*-test.

**
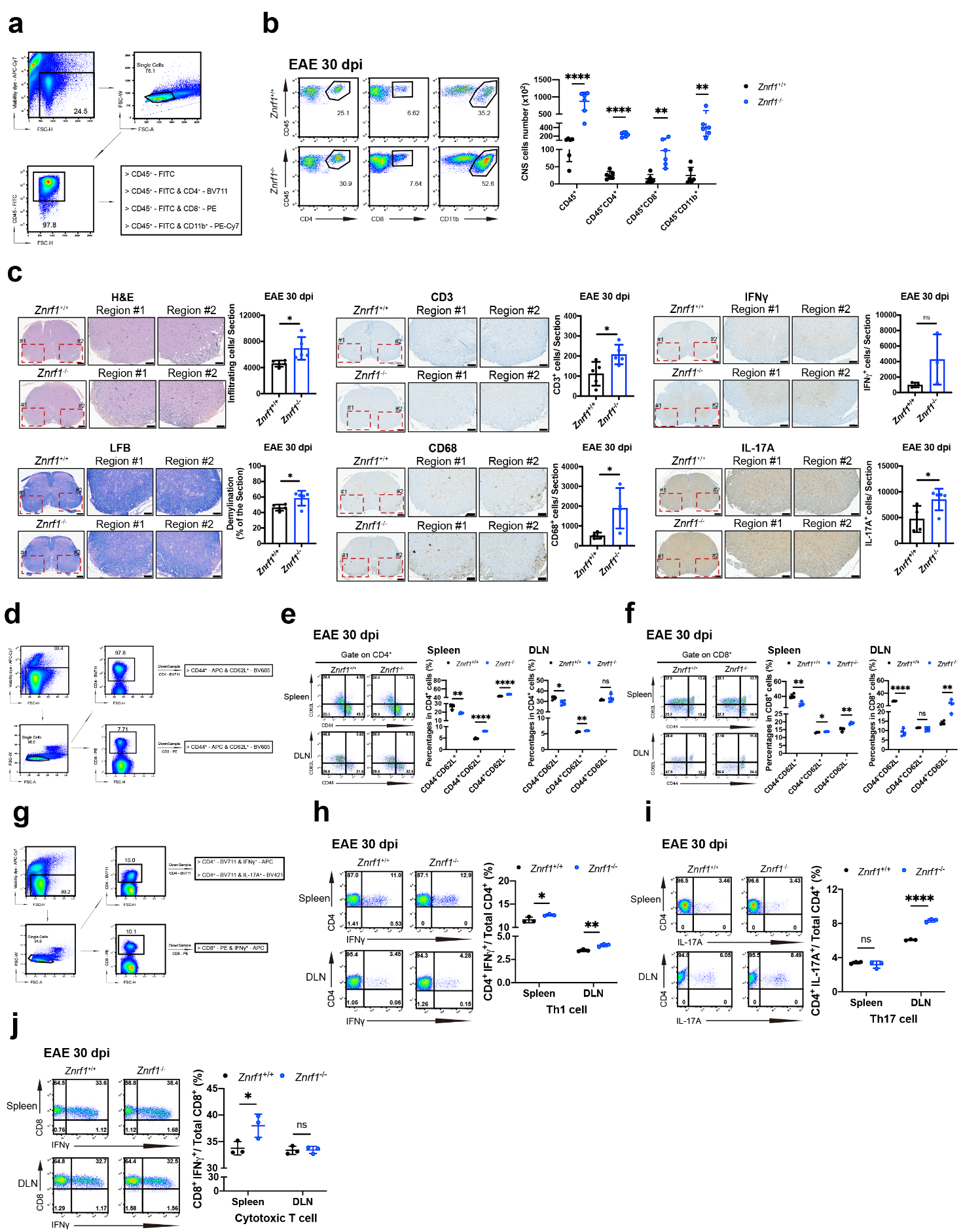
**

**Supplementary Figure 3. Loss of ZNRF1 increases immune cell infiltration in the spinal cord and alters T-cell differentiation during the late phase of EAE.**

(a) Gating strategy for identifying immune cell populations in the spinal cord. (b-j) Female *Znrf1*^+/+^ and *Znrf1*^-/-^ mice were sacrificed at day 30 after EAE induction. (b) Flow cytometric analysis of leucocytes (CD45^+^), Th cells (CD45^+^CD4^+^), cytotoxic T cells (CD45^+^CD8^+^), and myeloid cells (CD45^+^CD11b^+^) in single-cell suspensions from spinal cords (N=6). (c) Spinal cord sections from *Znrf1*^+/+^ and *Znrf1*^-/-^ mice were collected and analyzed at day 30 post-EAE induction. Tissue sections were subjected to H&E and LFB staining, as well as IHC using antibodies against CD3 (T cells), CD68 (macrophages), IFNγ (Th1 cells), and IL-17A (Th17 cells). Immune cell infiltration was quantified by IHC. Scale bars: 200 μm (whole spinal cord sections) and 100 μm (enlarged region #1 and #2). (d) Representative gating strategy for CD4^+^ and CD8^+^ T cell subsets. (e) Flow cytometric analysis of naïve (CD44^-^CD62L^+^), central memory (CD44^+^CD62L^+^), and effector memory (CD44^+^CD62L^-^) CD4^+^ T cells in spleens and DLNs of *Znrf1*^+/+^ (N=4) and *Znrf1*^-/-^ mice (N=4) at day 30 post-EAE induction. (f) Flow cytometric analysis of naïve (CD44^-^CD62L^+^), central memory (CD44^+^CD62L^+^), and effector memory (CD44^+^CD62L^-^) CD8^+^ T cells in spleens and DLNs of *Znrf1*^+/+^ (N=4) and *Znrf1*^-/-^ mice (N=4) at day 30 post-EAE induction. (g) Representative gating strategy for Th cell subsets (Th1 and Th17) and cytotoxic T cells. (h-j) Flow cytometric analysis of Th1 cells (CD4^+^IFNγ^+^) (h), Th17 cells (CD4^+^IL-17A^+^) (i), and cytotoxic T cells (CD8^+^IFNγ^+^) (j) in spleens and DLNs of *Znrf1*^+/+^ (N=3) and *Znrf1*^-/-^ mice (N=3) at day 30 post-EAE induction. (b) Representative flow plots and quantification of infiltrating immune cells in the CNS. (c) Representative spinal cord sections and quantified immune cells in the spinal cord of *Znrf1*^+/+^ and *Znrf1*^-/-^ mice. (d-j) Representative flow plots and quantified T cell populations in the spleen and DLNs. Data are presented as mean ± SD. ns, not significant. **P* < 0.05, ***P* < 0.01, ****P* < 0.001, *****P* < 0.0001, determined by the unpaired Student’s *t*-test.

**
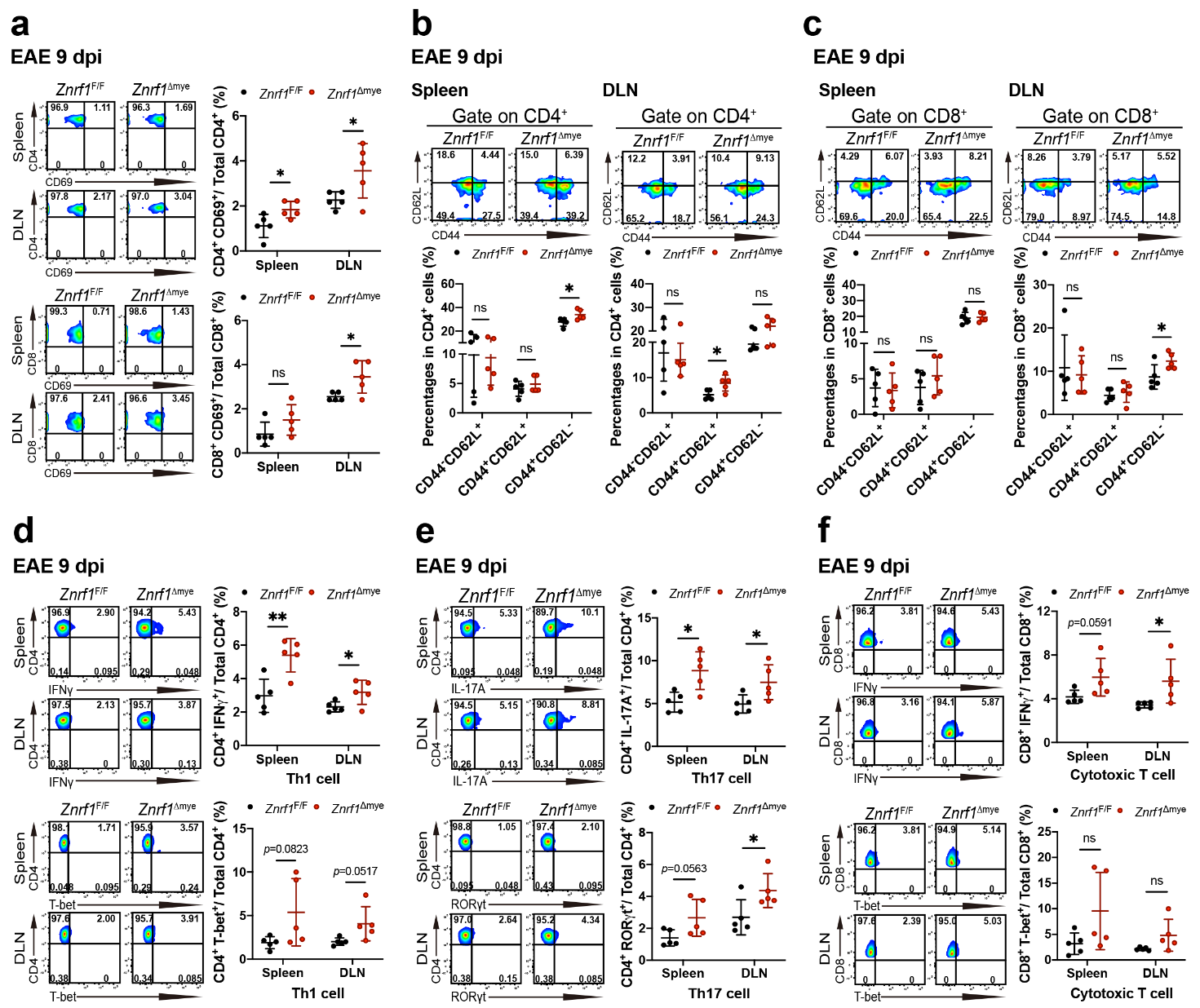
**

**Supplementary Figure 4. ZNRF1 deficiency in myeloid cells enhances T cell differentiation in peripheral lymphoid organs during the early phase of EAE induction.**

(a) Flow cytometric analysis of activated CD4^+^ T cells (CD4^+^CD69^+^) and CD8^+^ T cells (CD8^+^CD69^+^) in the spleen and DLNs of *Znrf1*^F/F^ (N=5) and *Znrf1*^Δmye^ mice (N=5) at day 9 post-EAE induction. (b) Flow cytometric analysis of naïve CD4^+^ T cells (CD4⁺CD44⁻CD62L⁺), CD4^+^ T_CM_ cells (CD4⁺CD44^+^CD62L⁺), and CD4^+^ T_EM_ cells (CD4⁺CD44^+^CD62L^-^) in spleens and DLNs of *Znrf1*^F/F^ (N=5) and *Znrf1*^Δmye^ mice (N=5) at day 9 post-EAE induction. (c) Flow cytometric analysis of naïve CD8^+^ T cells (CD8⁺CD44⁻CD62L⁺), CD8^+^ T_CM_ cells (CD8⁺CD44^+^CD62L⁺), and effector memory CD8^+^ T_EM_ cells (CD8⁺CD44^+^CD62L^-^) in spleens and DLNs of *Znrf1*^F/F^ (N=5) and *Znrf1*^Δmye^ mice (N=5) at day 9 post-EAE induction. (d-f) Flow cytometric analysis of Th1 cells (CD4⁺IFNγ⁺ and CD4^+^T-bet^+^) (d), Th17 cells (CD4⁺IL-17A⁺ and CD4^+^RORγt^+^) (e), and cytotoxic T cells (CD8^+^IFNγ^+^and CD8^+^T-bet^+^) (f) in spleens and DLNs of *Znrf1*^F/F^ (N=5) and *Znrf1*^Δmye^ mice (N=5) at day 9 post-EAE induction. Representative flow cytometry plots and quantification of T cell populations in spleens and DLNs are shown. Data are displayed as mean ± SD. ns, not significant. **P* < 0.05, ***P* < 0.01, determined by the unpaired Student’s *t*-test.


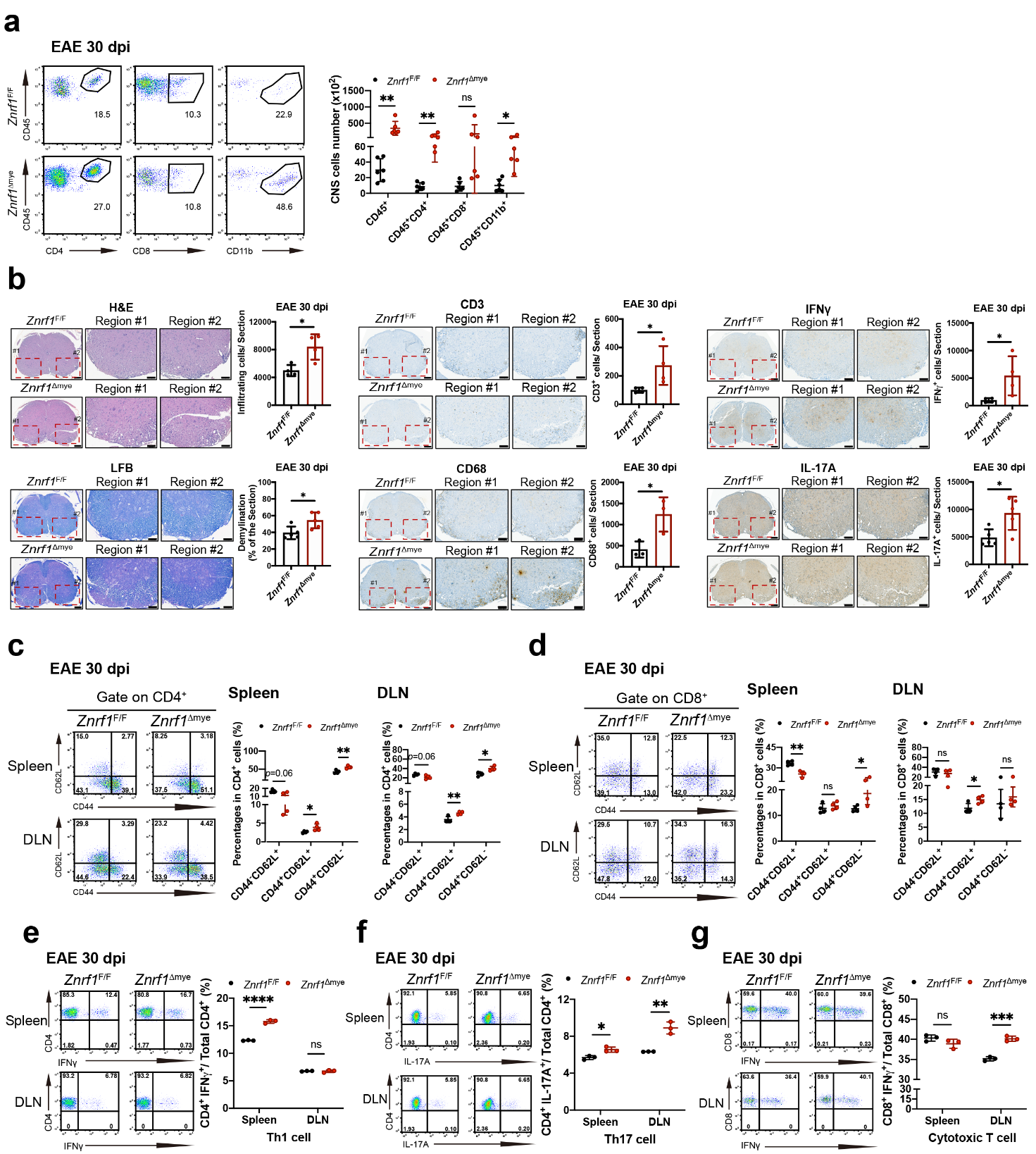


**Supplementary Figure 5. ZNRF1 deficiency in myeloid cells exacerbates EAE during the late phase.**

(a) Flow cytometric analysis of spinal cord single-cell suspensions from female *Znrf1*^F/F^ and *Znrf1*^Δmye^ mice at days 30 post-EAE induction. Cells were stained for CD45 (leucocytes), CD4 (Th cells), CD8 (cytotoxic T cells), and CD11b (myeloid cells) (N=6). Representative flow cytometry plots and quantification of infiltrating immune cells in the CNS are shown. (b) Histological and immunohistochemical analysis of spinal cord sections from *Znrf1*^F/F^ and *Znrf1*^Δmye^ mice at day 30 post-EAE induction. Tissue sections were stained with H&E and LFB, and immunostained with antibodies against CD3 (T cells), CD68 (macrophages), IFNγ (Th1 cells) and IL-17A (Th1 cells). Scale bars: 200 μm (whole spinal cord sections) and 100 μm (enlarged region #1 and #2). (c) Flow cytometric analysis of naïve (CD44^-^CD62L^+^), central memory (CD44^+^CD62L^+^), and effector memory (CD44^+^CD62L^-^) CD4^+^ T cells in the spleens and DLNs of *Znrf1*^F/F^ (N=4) and *Znrf1*^Δmye^ mice (N=4) at day 30 post-EAE induction. (d) Flow cytometric analysis of naïve, central memory, and effector memory CD8^+^ T cells in the spleens and DLNs of *Znrf1*^F/F^ (N=4) and *Znrf1*^Δmye^ mice (N=4) at day 30 post-EAE induction. (e-g) Flow cytometric analysis of Th1 cells (CD4^+^IFNγ^+^) (e), Th17 cells (CD4^+^IL-17A^+^) (f), and cytotoxic T cells (CD8^+^IFNγ^+^) (g) in the spleens and DLNs of *Znrf1*^F/F^ (N=3) and *Znrf1*^Δmye^ mice (N=3) at day 30 post-EAE induction. (a) Representative flow plots and quantification of CNS-infiltrating immune cells (a), spinal cord sections and quantified cells in spinal cords (b), and quantification of T cell subsets in spleens and DLNs (c-g). Data are mean ± SD. ns, not significant. **P* < 0.05, ***P* < 0.01, ****P* < 0.001, *****P* < 0.0001, determined by the unpaired Student’s *t*-test.

**
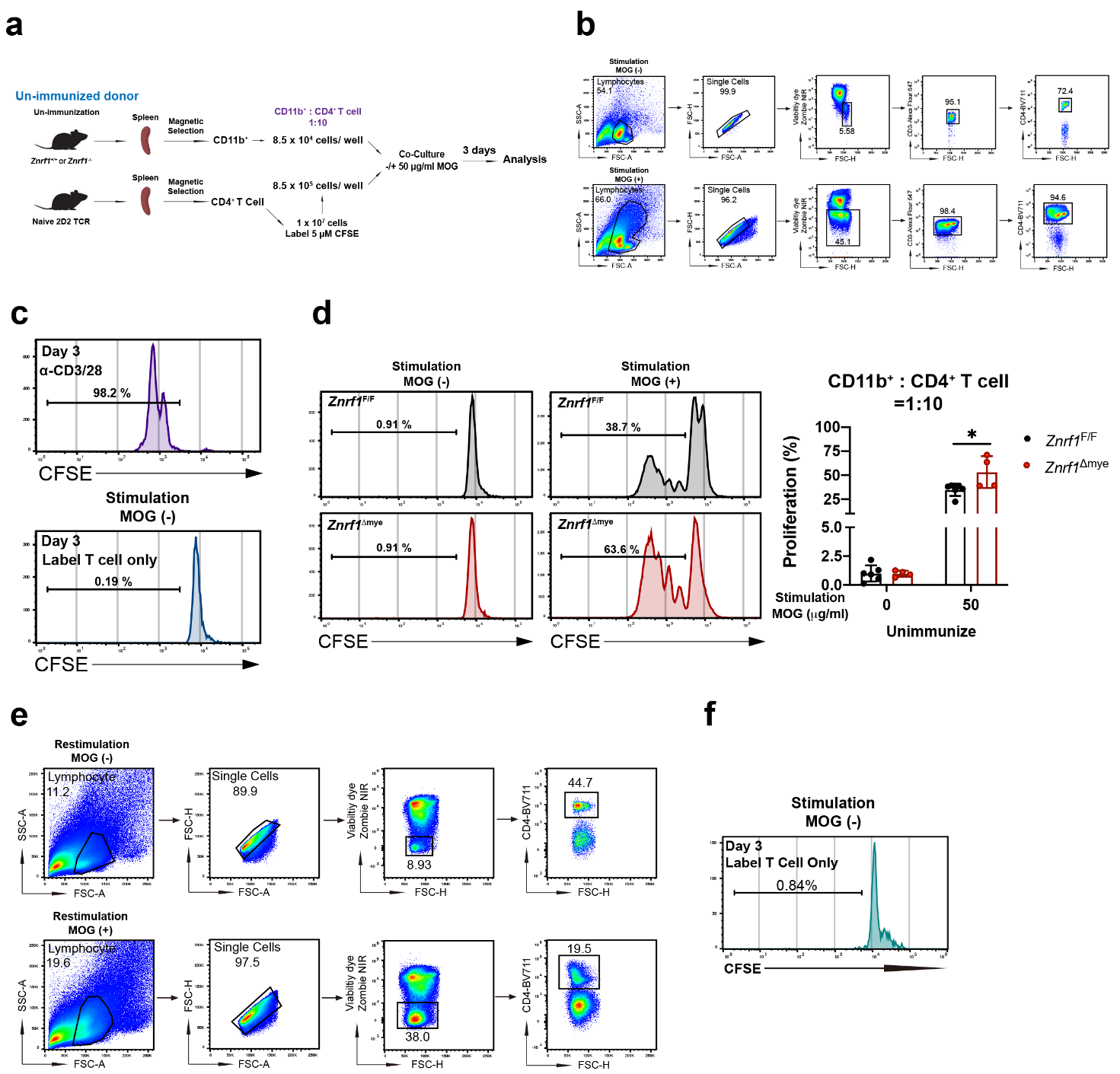
**

**Supplementary Figure 6. ZNRF1 deficiency in myeloid cells enhances antigen-dependent proliferation CD4^+^ T cells.**

(a) Schematic diagram of the experimental setup. CD11b^+^ cells were isolated from spleens of un-immunized *Znrf1*^F/F^ and *Znrf1*^Δmye^ mice using magnetic selection. CD4^+^ T cells were isolated from spleens of naive 2D2 mice via magnetic selection, labeled with 5 μM CFSE, and co-cultured with CD11b^+^ cells at a 1:10 ratio in the absence or presence of 50 μg/mL MOG_35–55_ for 3 days, followed by flow cytometry analysis. (b) Gating strategy for identifying proliferating CD4^+^ cells co-cultured with CD11b^+^ cells from unimmunized mice. (c) Representative flow cytometry plots of CFSE-labeled 2D2 CD4^+^ T cells stimulated with or without plate-bound anti-CD3 and soluble anti-CD28 antibodies for 3 days, serving as positive and negative controls for proliferation. (d) Flow cytometric analysis of CD4^+^ T cell proliferation in co-cultures of CD11b^+^ cells from un-immunized *Znrf1*^F/F^ (N=6) and *Znrf1*^Δmye^ (N=4) mice with CFSE-labeled 2D2 CD4^+^ T cells for 3 days, either in the absence or presence of 50 µg/ml MOG_35-55_. Representative flow cytometry plots (left) and quantification of proliferating CD4^+^ T cells (right) are shown. (e) Gating strategy for identifying proliferating CFSE-labeled CD4^+^ T cells co-cultured with CD11b^+^ cells from immunized mice. (f) Representative flow cytometry plot of CFSE-labeled 2D2 CD4^+^ T cells cultured alone for 3 days, serving as the negative control for proliferation. Data are presented as mean ± SD. ns, not significant. **P* < 0.05, determined by the unpaired Student’s *t*-test.


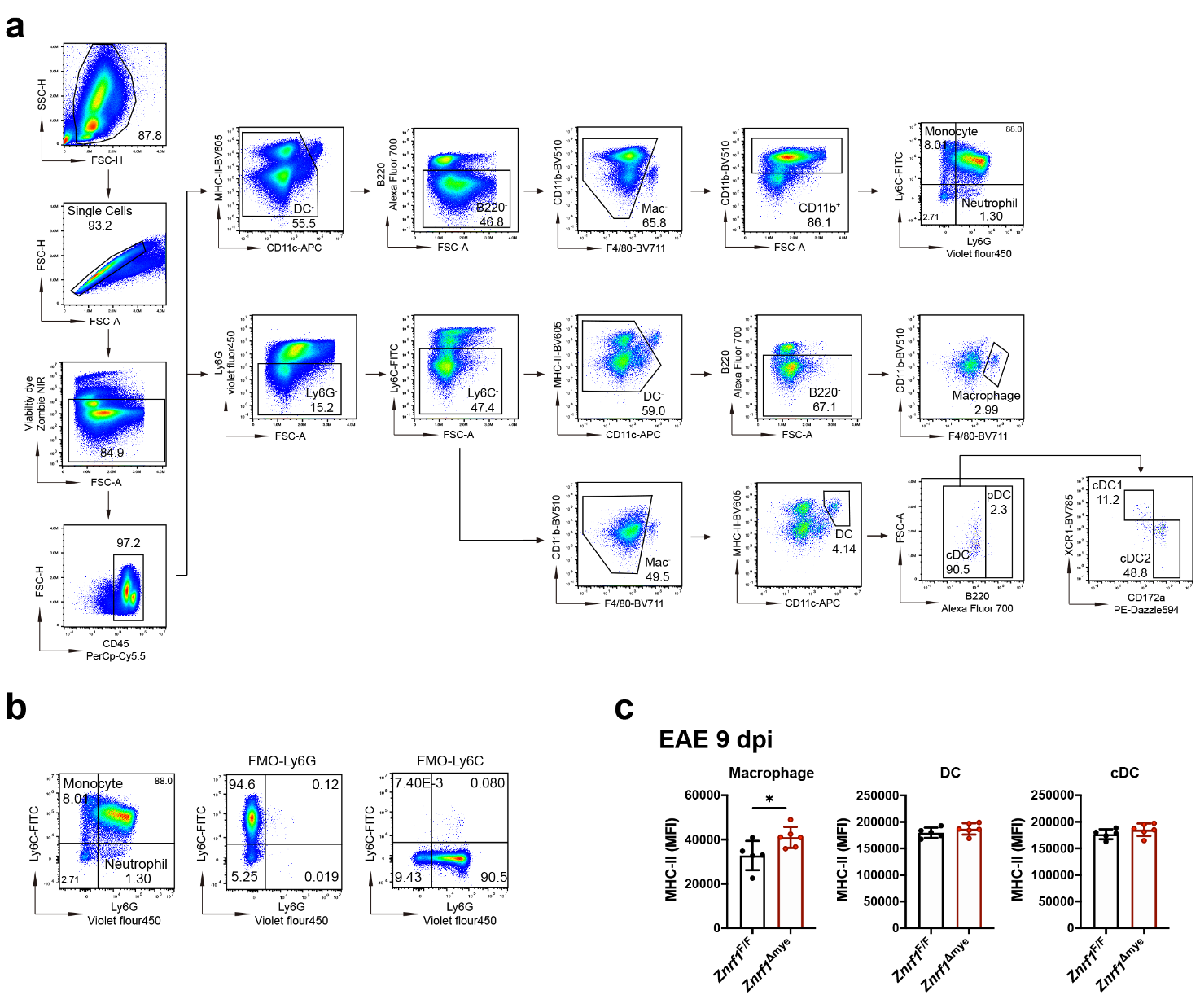


**Supplementary Figure 7. ZNRF1 deficiency in myeloid cells alters surface MHC-II expression on macrophages following EAE induction.**

(a) Flow cytometric gating strategy for identifying neutrophils (CD45^+^CD11c^-^B220^-^F4/80^-^CD11b^+^Ly6G^+^Ly6C^-^), monocytes (CD45^+^CD11c^-^B220^-^F4/80^-^CD11b^+^Ly6G^-^Ly6C^+^), macrophages (CD45^+^Ly6G^-^Ly6C^-^CD11c^-^B220^-^CD11b^+^F4/80^+^), dendric cells (DCs; CD45^+^Ly6G^-^Ly6C^-^CD11b^-^F4/80^-^CD11c^+^), conventional DCs (cDC; CD45^+^Ly6G^-^Ly6C^-^CD11b^-^F4/80^-^CD11c^+^B220^-^), plasmacytoid DCs (pDC; CD45^+^Ly6G^-^Ly6C^-^CD11b^-^F4/80^-^CD11c^+^B220^+^), cDC1 (CD45^+^Ly6G^-^Ly6C^-^CD11b^-^F4/80^-^CD11c^+^B220^-^CD172a^-^XCR1^+^), and cDC2 (CD45^+^Ly6G^-^Ly6C^-^CD11b^-^F4/80^-^ CD11c^+^B220^-^CD172a^+^XCR1^-^) in spleens of immunized mice at day 12 post-EAE induction. (b) Fluorescence minus one (FMO) controls for Ly6G and Ly6C used to identify neutrophils and monocytes. (c) Flow cytometric analysis of macrophages, DCs, and cDCs in spleens of *Znrf1*^F/F^ (N=6) and *Znrf1*^Δmye^ (N=6) mice at day 9 post-EAE induction. The MFI of MHC-II expression was quantified. Data are presented as mean ± SD. ns, not significant. **P* < 0.05, determined by the unpaired Student’s *t*-test.


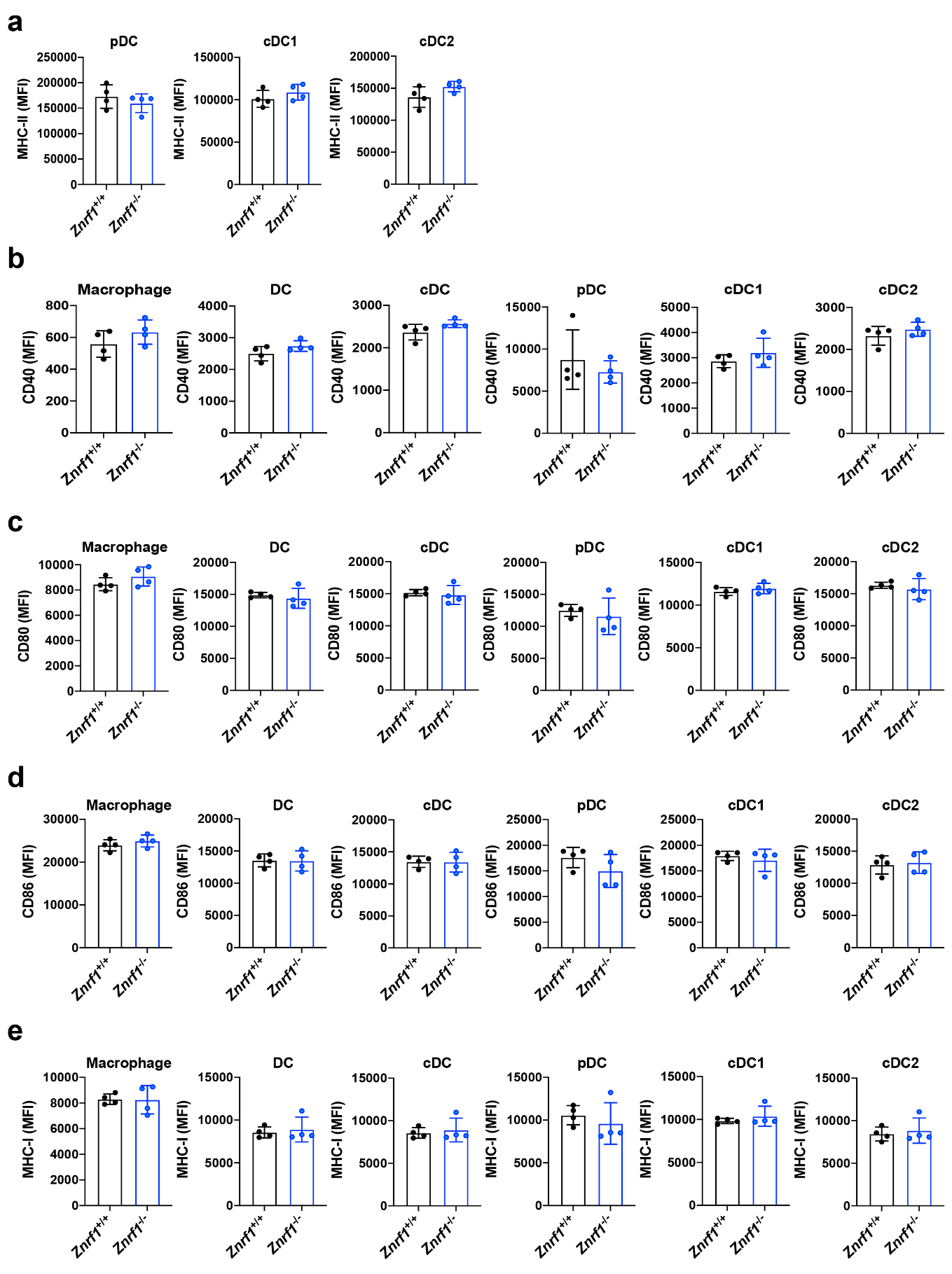


**Supplementary Figure 8. Systemic ZNRF1 deletion does not affect the expression of co-stimulatory surface markers on myeloid cells in the spleens after EAE induction.**

(a) Flow cytometric analysis of pDCs, cDC1s, and cDC2s in the spleens of *Znrf1*^+/+^ (N=4) and *Znrf1*^-/-^ (N=4) mice at day 12 post-EAE induction. The MFI of MHC-II was quantified. (b-e) Flow cytometric analysis of macrophages, DCs, cDCs, pDCs, cDC1s, and cDC2s in the spleens of *Znrf1*^+/+^ (N=4) and *Znrf1*^-/-^ (N=4) mice at day 12 post-EAE induction. The MFIs of CD40 (b), CD80 (c), CD86 (d), and MHC-I (e) were quantified. Data are presented as mean ± SD. ns, not significant. Statistical significance was determined by the unpaired Student’s *t*-test.


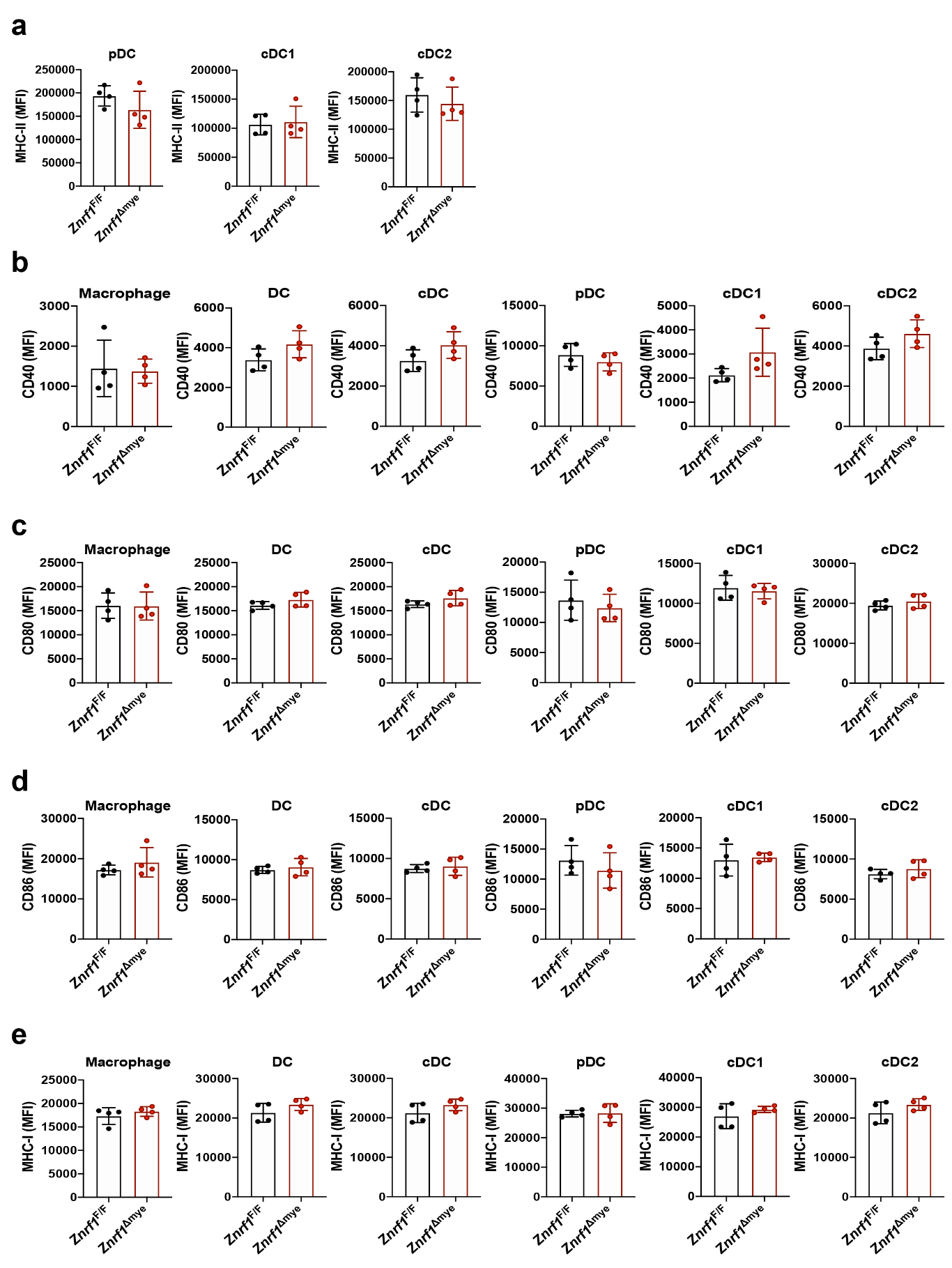


**Supplementary Figure 9. ZNRF1 deficiency in myeloid cells does not affect the expression of co-stimulatory surface markers on myeloid cells in the spleens after EAE induction.**

(a) Flow cytometric analysis of pDCs, cDC1s, and cDC2s in the spleens of *Znrf1*^F/F^ (N=4) and *Znrf1*^Δmye^ (N=4) mice at day 12 post-EAE induction. The MFI of MHC-II was quantified. (b-e) Flow cytometric analysis of macrophages, DCs, cDCs, pDCs, cDC1s, and cDC2s in the spleens of *Znrf1*^F/F^ (N=4) and *Znrf1*^Δmye^ (N=4) mice at day 12 post-EAE induction. The MFIs of CD40 (b), CD80 (c), CD86 (d), and MHC-I (e) were quantified. Data are presented as mean ± SD. ns, not significant. Statistical significance was determined by the unpaired Student’s *t*-test.


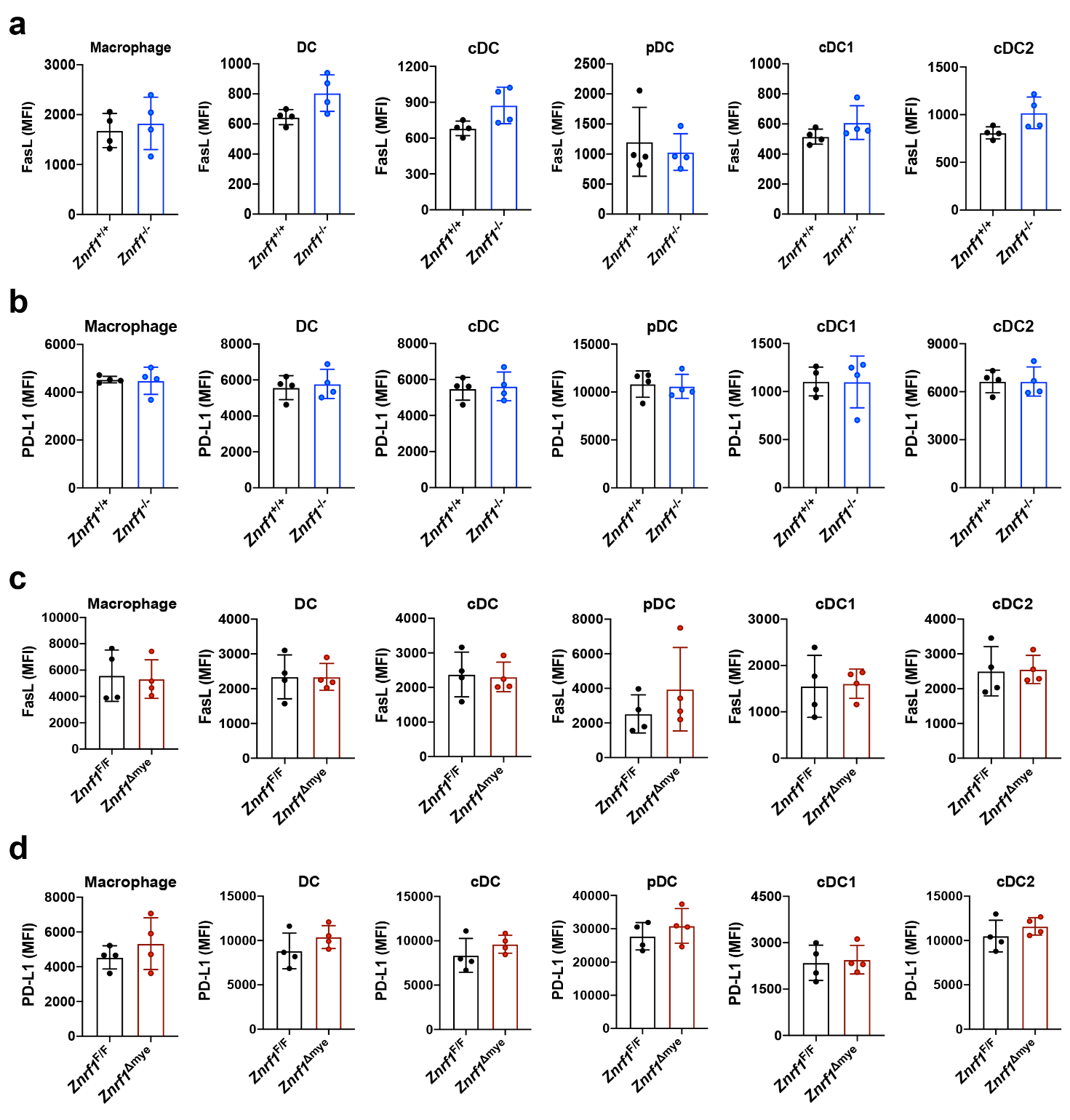


**Supplementary Figure 10. ZNRF1 deficiency does not affect the expression of surface inhibitory markers on myeloid cells in the spleens after EAE induction.**

(a, b) Flow cytometric analysis of macrophages, DCs, cDCs, pDCs, cDC1s, and cDC2s in the spleens of *Znrf1*^+/+^ (N=4) and *Znrf1*^-/-^ (N=4) mice at day 12 post-EAE induction. The MFIs of FasL (a) and PD-L1 (b) were quantified. (c-d) Flow cytometric analysis of macrophages, DCs, cDCs, pDCs, cDC1s, and cDC2s in the spleens of *Znrf1*^F/F^ (N=4) and *Znrf1*^Δmye^ (N=4) mice at day 12 post-EAE induction. The MFIs of FasL (c) and PD-L1 (d) were quantified. Data are presented as mean ± SD. ns, not significant. Statistical significance was determined by the unpaired Student’s *t*-test.

**Supplementary Table 1**

The primers used for mouse genotyping are listed below.

| **Primer name** | **Primer Sequence (5’ to 3’)** |
| --- | --- |
| Znrf1_Flox_Forward | AACATCTCAGGAAGCCACTAAC |
| Znrf1_Flox_Reverse | GAAAAGGAAAACTAAAACATCG |
| Znrf1_Flox_Forward 2 | TGACTAGTCGTCGTCCCCCTTTTT |
| LysM_ wild type | TTACAGTCGGCCAGGCTGAC |
| LysM_common | CTTGGGCTGCCAGAATTTCTC |
| LysM_Mutant | CCCAGAAATGCCAGATTACG |
| Cx3Cr1_Cre/ERT_EYFP_Common | AAGACTCACGTGGACCTGCT |
| Cx3Cr1_Cre/ERT_EYFP_Mutant Reverse | CGGTTATTCAACTTGCACCA |
| Cx3Cr1_Cre/ERT_EYFP_Wild type Reverse | AGGATGTTGACTTCCGAGTTG |
| MxCre_Forward | ACCTGAAGATGTTCGCGATTATCT |
| MxCre_Reverse | ACCGTCAGTACGTGAGATATCTT |
| Cre1_Forword | GCCTGCATTACCGGTCGATGCAACGA |
| Cre1_Reverse | GTGGCAGATGGCGCGGCAACACCATT |


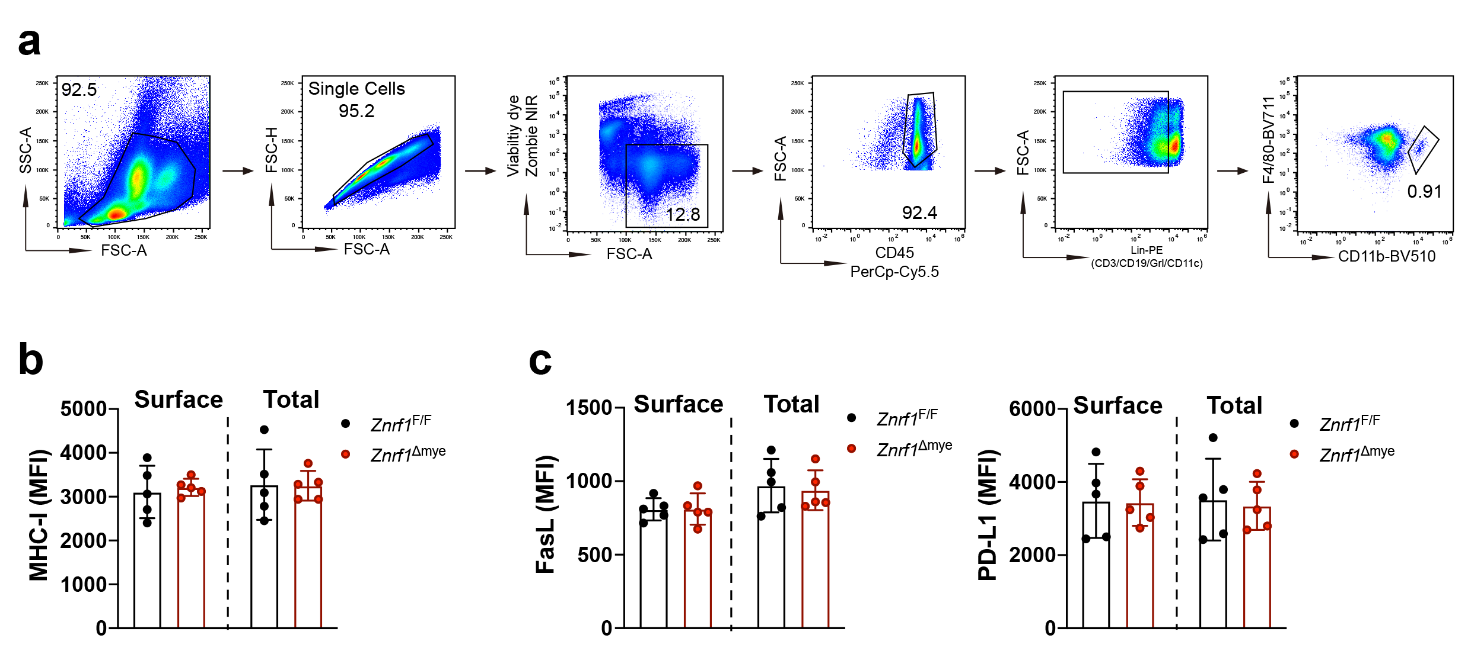


**Supplementary Figure 11. ZNRF1 deficiency does not alter surface or total expression of MHC-I, FasL, or PD-L1 on splenic myeloid cells after EAE induction.**

(a) Flow cytometric gating strategy for identifying macrophages (CD45^+^CD3^-^CD19^-^Gr1^-^CD11c^-^CD11b^+^F4/80^+^) in spleens of immunized mice at day 12 post-EAE induction. (b-c) Quantification of surface and total MFIs of MHC-I (b), FasL (c) and PD-L1 (c). Data are presented as mean ± SD. ns, not significant. Statistical significance was determined by the unpaired Student’s *t*-test.
